# Aberrant VEGFR2 supports tumor growth by extracellular matrix remodeling

**DOI:** 10.1038/s41419-025-08404-3

**Published:** 2026-01-15

**Authors:** Michela Corsini, Cosetta Ravelli, Mattia Domenichini, Anna Ventura, Camilla Maggi, Elisa Moreschi, Mirko Tamma, Chiara Romani, Claudia Piccoli, Elisabetta Grillo, Stefania Mitola

**Affiliations:** 1 Department of Molecular and Translational Medicine, University of Brescia, Via Branze 39, Brescia, Italy; 2 The Mechanobiology Research Center,UNIBS, University of Brescia, Brescia, Italy; 3 Department of Clinical and Experimental Medicine, University of Foggia, Foggia, Italy; 4 Medical and Surgical Specialties, Radiological Sciences and Public Health, University of Brescia, Brescia, Italy; 5 Angelo Nocivelli Institute of Molecular Medicine, ASST Spedali Civili of Brescia, Brescia, Italy

**Keywords:** Cancer microenvironment, Extracellular matrix

## Abstract

The extracellular matrix shapes tumor architecture, cell behavior and therapy response. Here, we identify aberrant activation of the receptor tyrosine kinase VEGFR2 as a driver of tumor-promoting ECM remodeling in melanoma and ovarian cancer. ECM alterations in terms of composition and organization were observed in Sk-Mel-31 melanoma xenografts expressing the oncogenic VEGFR2^R1032Q^ and in ovarian tumors with VEGFR2 hyperactivation. Down-modulation of VEGFR2 normalized ECM architecture. Decellularized ECM from VEGFR2^R1032Q^ melanoma cells directly modified the behavior of VEGFR2^WT^ tumor cells, increasing monolayer fluidity and mitochondrial activation. Transcriptomic profiling revealed a dysregulation of genes involved in ECM structure and remodeling, mediated by the PI3K-AKT and ERK pathways. Pharmacological inhibition of VEGFR2 with tyrosine kinase inhibitors, such as lenvatinib, partially reverted ECM alterations in vitro and in vivo, reducing matrix deposition and modifying its organization. These data identify VEGFR2 as a regulator of tumor ECM dynamics and suggest that its inhibition may restore ECM organization, offering a therapeutic strategy to reprogram the tumor microenvironment and limit cancer progression.

## Introduction

Solid tumors are complex tissues comprising cancer cells and various components of the tumor microenvironment (TME). The TME includes resident and infiltrating cells, as well as extracellular matrix (ECM), which provides a structural scaffold for cells while dynamically influencing cancer growth, progression, and metastasis. Beyond defining tumor architecture, the ECM contributes to the mechanical properties of tumors, such as stiffness and elasticity, and serves as a reservoir of soluble factors, including growth factors [1, 2].

Both cancer and stromal cells maintain the ECM homeostasis through continuous deposition and remodeling. Aberrant ECM deposition and structural modifications contribute to the mechanical and biochemical properties of the tumor, fostering a pro-tumorigenic microenvironment [3]. Alterations in ECM composition and organization are increasingly recognized as drivers of tumor progression and metastasis, with enhanced ECM deposition, or desmoplasia, often correlating with poor clinical outcomes in cancers such as colorectal, renal, cervical, lung, and breast cancers [4–6].

Vascular Endothelial Growth Factor Receptor 2 (VEGFR2) is a classical tyrosine kinase receptor involved in tumor vascularization. However, increasing evidence highlights its pro-oncogenic effects independent of its pro-angiogenic role. VEGFR2 regulates cancer cell proliferation, migration, and metabolic adaptation [7].

An aberrant VEGFR2 activation has been described in different cancers, including colon cancer and melanoma [8, 9]. Pan cancer analysis suggests that this activation can result from somatic mutations and gene amplifications in VEGFR2 gene highlighting these alterations in melanoma samples, as well as from the ligand dysregulation such as the overexpression of VEGF in ovarian cancer [9–11]. Despite these observations, the direct involvement of tumor cell-intrinsic VEGFR2 in ECM remodeling and its implications for cancer progression remain largely unexplored.

In this study, we investigate how aberrant VEGFR2 activation in tumor cells, through the expression of an oncogenic mutant or dysregulation of receptor levels, impacts on ECM composition and dynamics in melanoma and ovarian cancer models. We demonstrate that VEGFR2 in the tumor regulates tumor ECM remodeling, contributing to an altered tumor microenvironment that promotes tissue fluidity, tumor growth, and progression, promoting alterations that support at least in part tumor growth.

These effects are mediated, at least in part, through ERK/MAPK signaling, a key pathway downstream of VEGFR2 activation. Furthermore, we identify that VEGFR2-targeting tyrosine kinase inhibitors, such as lenvatinib, at least partially reversing these ECM alterations, reducing its deposition and restoring its structural organization both in vitro and in vivo.

## Results

### Aberrant activation of VEGFR2 drives the remodeling of tumor extracellular matrix

**Fig. 1:**
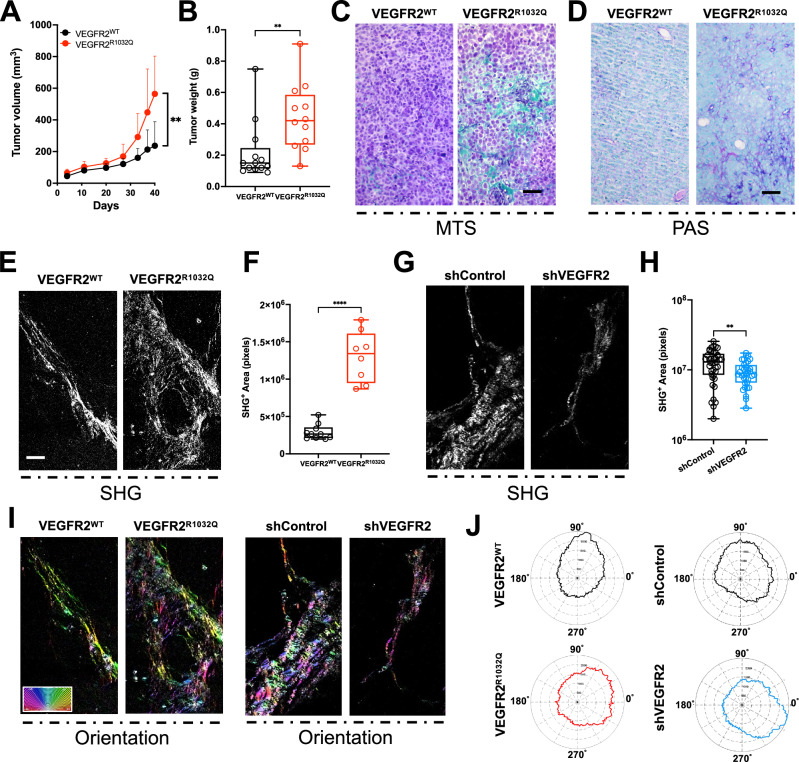
Collagen fibers lost their orderly arrangement in the VEGFR2 hyperactive tumor. **A** Tumor growth curve of VEGFR2^WT^ and VEGFR2^R1032Q^ Sk-Mel-31-derived tumor. Data are presented as the mean ± standard deviation (*n* = 11–12). Statistical significance was determined by mixed-effects analysis with uncorrected Fisher’s LSD, **P < 0.01. **B** Tumor weight of VEGFR2^WT^ and VEGFR2^R1032Q^ Sk-Mel-31-derived tumor. Single data are represented as empty dots in Min to Max box & whiskers plot (*n* = 12–13). Statistical significance was determined by Unpaired *t* test, **P < 0.01. **C** Representative MTS staining of FFPE slices from VEGFR2^WT^ and VEGFR2^R1032Q^ Sk-Mel-31-derived tumors (n = 5). Scale bars, 50 µm. **D** Representative PAS staining of FFPE slices from VEGFR2^WT^ and VEGFR2^R1032Q^ Sk-Mel-31-derived tumors. Scale bars, 50 µm. **E** Second harmonic generation (SHG) imaging of collagen fibers in FFPE slices from VEGFR2^WT^ and VEGFR2^R1032Q^ Sk-Mel-31-derived tumors. Scale bars, 50 µm. **F** Quantification of SHG^+^ area in VEGFR2^WT^ and VEGFR2^R1032Q^ Sk-Mel-31-derived tumors. Single data are represented as empty dots in Min to Max box & whiskers plot (*n* = 8–10). Statistical significance was determined by two-way ANOVA ***P < 0.001. **G** Second harmonic generation (SHG) imaging of collagen fibers in FFPE slices from shControl and shVEGFR2 OVCAR3-derived tumors. Scale bars, 50 µm. **H** Quantification of SHG^+^ area in shControl and shVEGFR2 OVCAR3-derived tumors. Single data are represented as empty dots in Min to Max box & whiskers plot (*n* = 25–35). Statistical significance was determined by two-way ANOVA, **P < 0.01. **I** Collagen fiber orientation maps of FFPE slices from VEGFR2^WT^ and VEGFR2^R1032Q^ Sk-Mel-31-derived tumors. Color coding indicates fiber orientation. Scale bars, 50 µm. **J** Representative distribution of collagen fiber orientation angles from SHG images in VEGFR2^WT^ and VEGFR2^R1032Q^ Sk-Mel-31-derived tumors and from shControl and shVEGFR2 OVCAR3-derived tumors in polar plot coordinates.

Recently we showed that the presence of substitution R1032Q in VEGFR2 induced receptor aberrant activation and enhanced tumor growth [8, 12]. To deepen the effects of this hyperactivation VEGFR2^WT^ or VEGFR2^R1032Q^ receptors were stably expressed in Sk-Mel-31 melanoma cells and subcutaneously injected in NOD/SCID mice. The expression of VEGFR2^R1032Q^ resulted in a significantly faster Sk-Mel-31 tumor growth (Fig. 1A), with a mean tumor volume measured at the endpoint of 237.1 ± 151.3 mm^3^ in VEGFR2^WT^ Sk-Mel-31-derived tumors compared to 564.3 ± 238.9 mm^3^ in VEGFR2^R1032Q^ Sk-Mel-31 group. This data was further corroborated by increased tumor weight at 40 days (Fig. 1B), confirming enhanced tumorigenic potential in a human skin melanoma model.

Histological analysis of tumor sections revealed distinct differences in tumor architecture. Masson’s Trichrome Stain (MTS) highlighted prominent collagen-rich fibrotic regions in VEGFR2^R1032Q^-derived tumors compared to VEGFR2^WT^ samples. Notably, VEGFR2^R1032Q^Sk-Mel-31-derived tumors exhibited irregular, variable-sized nest-like structures surrounded by extracellular matrix (ECM), whereas VEGFR2^WT^ tumors displayed smaller, more uniform nests. (Fig. 1C). In skin melanoma, the presence of nest-like structures is often associated with rapid ECM remodeling [13]. Periodic acid-Schiff (PAS) staining further confirmed the higher accumulation of collagen, polysaccharides, and other matrix components in VEGFR2^R1032Q^ expressing tumors (Fig. 1D).

To provide a more detailed characterization of the ECM, we performed label-free second harmonic generation (SHG) imaging on paraffin-embedded (FFPE) tissue sections. Quantitative analysis of SHG-positive areas indicated a greater signal in VEGFR2^R1032Q^-derived tumor sections compared to VEGFR2^WT^ (Fig. 1E, F). This induced us to investigate the ECM remodeling in an alternative VEGFR2-dependent cancer model. To this we analyzed ovarian cancer (OVCAR3) cell tumor-derived in which VEGFR2 was silenced by shRNA lentiviral particles. Of note, in OVCAR3-derived tumors, VEGFR2 phosphorylation was high (Supplementary Fig. 1A) with a parallel abundance of ECM as demonstrated by H&E and Masson’s staining (Supplementary Fig. 1B, C).

As anticipated, the VEGFR2 silencing reduced the SHG-positive area in xenograft tumors (Fig. 1G, H). Then, we investigated the fibril orientation in ECM in Sk-Mel-31 and OVCAR3-derived tumor sections using the OrientationJ plugin of ImageJ, which provide a visual representation of fibril orientation distribution within the ECM [14]. The color-coded angle distribution (Fig. 1I), along with the polar coordinate representations (Fig. 1J), confirmed a disorganized distribution of fiber pattern and a significant difference in ECM orientation in tumors with aberrant VEGFR2-activations.

**Fig. 2:**
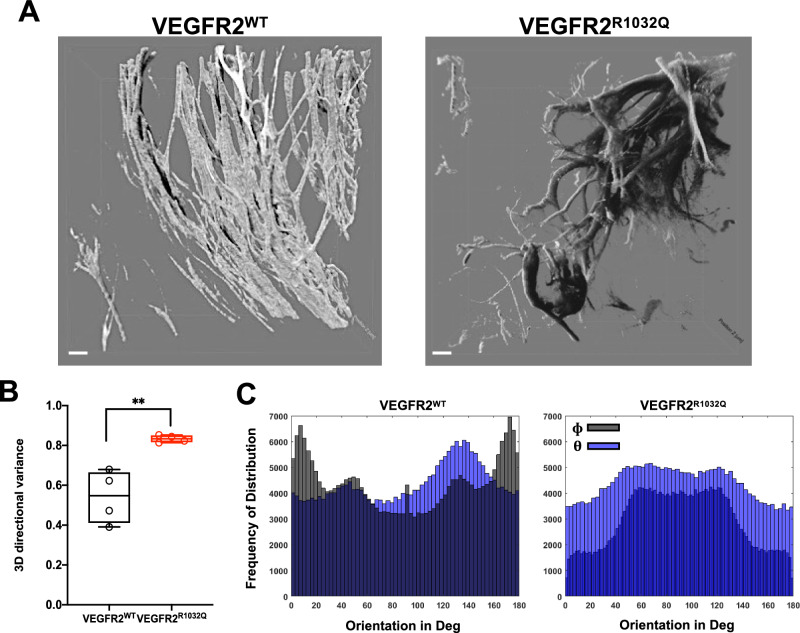
Collagen fibers reorganized random in VEGFR2^R1032Q^ Sk-Mel-31 derived tumors. **A** Representative 3D renderings of collagen organization using SHG analysis in VEGFR2^WT^ and VEGFR2^R1032Q^ Sk-Mel-31-derived tumors. Scale bars 20 µm (*n* = 4). **B** Quantification of 3D directional variance of collagen orientation. Single data are represented as empty dots in Min to Max box & whiskers plot (*n* = 4). **C** Representative frequency distribution of collagen orientation angles (Φ and Θ) in VEGFR2^WT^ and VEGFR2^R1032Q^ tumors (*n* = 4). Statistical significance was determined by two-way ANOVA, **P < 0.01.

To reconstruct 3D tissue structure SHG analysis was performed on optically cleared Sk-Mel-31-derived tumors (Fig. 2A). Collagen fibers orientation was quantified using a MATLAB script calculating the variance index and azimuthal angles [15].

Consistent with the 2D findings, VEGFR2^R1032Q^ Sk-Mel-31-derived tumors exhibited higher variance in 3D spatial topology, reflecting a more random ECM arrangement (Fig. 2B). In line with this data, the distribution of azimuthal angles φ and θ was more widely distributed in SK-Mel-31-VEGFR2^R1032Q^ samples (Fig. 2C).

### Expression of VEGFR2^R1032Q^ alters the gene expression profile of melanoma

**Fig. 3:**
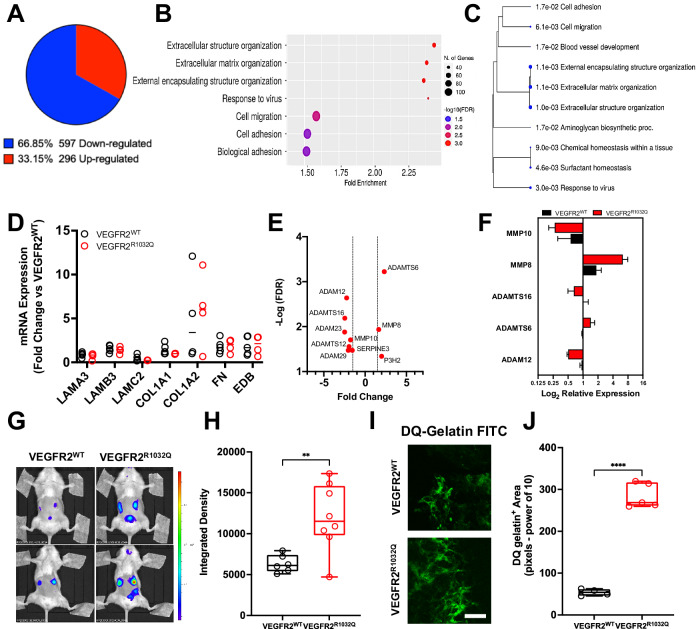
Transcriptomic and functional analysis of ECM remodeling in VEGFR2^WT^ and VEGFR2^R1032Q^ Sk-Mel-31-derived tumors. **A** Pie chart of differentially expressed genes in VEGFR2^R1032Q^ Sk-Mel-31-derived tumors vs VEGFR2^WT^, upregulated (red) and downregulated (blue) genes (*n* = 4). **B** Gene ontology (GO) enrichment analysis of differentially expressed genes. Bubble size represents the number of genes associated with each term, and color indicates significance. **C** Hierarchical clustering of enriched biological processes. **D** mRNA expression levels of ECM components. Data are presented as fold change relative to VEGFR2^WT^ Sk-Mel-31-derived tumors. Data comes from *n* = 2 independent experiment and from *n* = 4–5 replicates. **E** Volcano plot of differentially expressed ECM-modifying enzymes. Dashed lines indicate significance thresholds. **F** Log2 relative expression of MMPs and ADAMTS proteases in VEGFR2^WT^ -and VEGFR2^R1032Q^ Sk-Mel-31-derived tumors. Data comes from *n* = 2 independent experiment and from *n* = 3 replicates. **G** In vivo imaging of proteolytic activity using MMPSense probes in tumor-bearing mice. **H** Quantification of fluorescence signal intensity. Single data are represented as empty dots in Min to Max box & whiskers plot (*n* = 6–8). Statistical significance was determined by two-way ANOVA, **P < 0.01. **I** Ex vivo imaging of proteolytic activity using DQ-gelatin probe. Scale bar, 50 µm. **J** Quantification of DQ-gelatin degradation area. Single data are represented as empty dots in Min to Max box & whiskers plot (*n* = 4–5). Statistical significance was determined by two-way ANOVA, ***P < 0.001.

To investigate the effects of VEGFR2^R1032Q^ expression on ECM dynamics, the transcriptomic profile of Sk-Mel-31 derived tumors were generated using Clariom_S Affymetrix microarray chips. The analysis identified 893 human genes differentially expressed in VEGFR2^R1032Q^ Sk-Mel-31-expressing tumors compared to those expressing VEGFR2^WT^. Among these, 296 genes (33.15%) were upregulated. In comparison, 597 genes (66.85%) were downregulated (Fig. 3A and Supplementary Table 1). Gene ontology (GO) enrichment analysis for biological processes, performed using ShinyGO 0.76, highlighted extracellular structure organization, extracellular matrix organization, and external encapsulating structure organization as the top-ranked enriched pathways. These pathways exhibited enrichment FDR values ranging from 0.000559 and 0.00404. The corresponding −Log (FDR) values for the top three enriched processes were 2.49, 2.43, 2.41, respectively, as illustrated in the lollipop chart (Fig. 3B) and the hierarchical clustering tree (Fig. 3C). While transcriptomic analysis did not reveal significant changes in the expression of structural ECM proteins, as confirmed by qPCR (Fig. 3D), several genes related to ECM dynamics were significantly modulated in VEGFR2^R1032Q^ Sk-Mel-31-derived tumors (Fig. 3E). Among the upregulated genes were ITGA11, ADAMTS6, and P3H2, which are involved in ECM remodeling. ADAMTS6 [16] encodes a disintegrin involved in protein metabolism and post-translational O-glycosylation of mucins, while P3H2 contributes to post-translational collagen IV modification [17], and ITGA11 plays a role in integrin-mediated collagen adhesion [18].

Conversely, several ECM-related genes were downregulated, including ADAM12, COL18A1, AGRN, MATN3, ITGB6, ITGB8, and TGFβ1. These genes are associated with structural ECM integrity and cell-matrix interactions. COL18A1 [19], AGRN [20] and MATN3 [21] play a role in ECM structure contributing to the formation of extracellular filamentous networks, while ITGB6 and ITGB8 mediate integrin-dependent cell-fibronectin interactions [22]. ADAM12 encodes a protease with a cysteine-rich domain involved in ECM remodeling and cell adhesion [23], whereas TGFβ1 plays a crucial regulatory role in ECM composition and fibrosis [24]. The expression of these ECM-related genes was validated by qPCR (Fig. 3F). Matrix remodeling activity was further investigated in vivo using the MMPsense 750 Fast Fluorescent Imaging Agent. Luminescence was significantly higher in VEGFR2^R1032Q^ Sk-Mel-31-derived tumors reflecting increased matrix remodeling activity (Fig. 3G, H). These results were corroborated ex vivo by probing tumor fragments with DQ-gelatin FITC, where from enzymatic cleavage of the substrate release fluorescence, further confirming matrix remodeling in VEGFR2^R1032Q^ Sk-Mel-31-derived tumors (Fig. 3I, J).

### ECM controls tissue fluidity

**Fig. 4:**
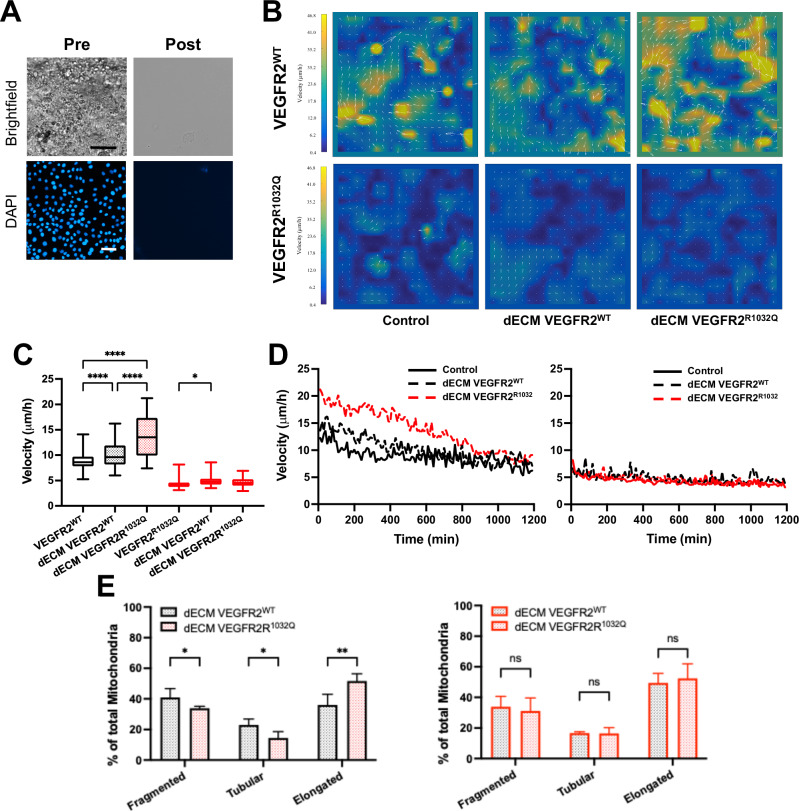
Decellularized ECM from VEGFR2^R1032Q^ cells impairs monolayer fluidity. **A** Brightfield images and DAPI stain of Sk-Mel-31 monolayer pre- and post-decellularization. **B** Velocity heatmaps with overlaid vector fields showing the direction and magnitude of monolayer fluidity of VEGFR2^WT^ (top row) and VEGFR2^R1032Q^ (bottom row) Sk-Mel-31 cells seeded on control substrates, dECM from VEGFR2^WT^ cells, or VEGFR2^R1032Q^ cells. Color scale indicates cell velocity (μm/h). **C** Quantification of monolayer fluidity average velocity. Data are represented in Min to Max box & whiskers plot (*n* = 400 cells) for *n* = 3 independent experiments. Statistical significance was determined by two-way ANOVA, *P < 0.05, ***P < 0.001, ****P < 0.0001. **D** Time course of average monolayer fluidity for VEGFR2^WT^ (left) and VEGFR2^R1032Q^ (right) Sk-Mel-31 cells migrating on control, dECM VEGFR2^WT^ cells, or VEGFR2^R1032Q^ cells substrates. Migration velocity was measured over an 18-h period (*n* = 3 independent experiments). **E** Mitochondrial networks of VEGFR2^WT^ (left) and VEGFR2^R1032Q^ (right) Sk-Mel-31 cells on dECM VEGFR2^WT^ cells, or VEGFR2^R1032Q^ cells substrates. Fragmented, tubular, and elongated mitochondria are represented as mean ± SD (*n* = 40–50 cells) for *n* = 2 independent experiments. Statistical significance was determined by two-way ANOVA, *P < 0.05, **P < 0.01.

To mimic the effects of a remodeled ECM on cancer cell behavior in vitro, we established a decellularization protocol to obtain ECM (dECM) produced by cancer cells in vitro (Fig. 4A). Since ECM remodeling influences tissue fluidity, the transition of tissue from a solid-like state to a fluid-like state, we analyzed the fluidity of confluent monolayers of VEGFR2^WT^ and VEGFR2^R1032Q^ Sk-Mel-31. To this, VEGFR2^WT^ Sk-Mel-31 monolayer cells, followed by time-lapse video microscopy for 16 h, displayed higher fluidity compared to the Sk-Mel-31 expressing mutated VEGFR2 (Fig. 4B–D). Next, the syngeneic Sk-Mel-31 cells were plated onto dECM derived from VEGFR^WT^ Sk-Mel-31 or VEGFR^R1032Q^ Sk-Mel-31 cells. Adhesion to dECM derived from VEGFR2^R1032Q^ SK-Mel-31 cells significantly increased the fluidity of VEGFR2^WT^ Sk-Mel-31 monolayer (Fig. 4B–D), while adhesion to dECM from VEGFR2^WT^ cells, did not alter their behavior. In contrast, the fluidity of VEGFR2^R1032Q^ Sk-Mel-31 cells remained unchanged regardless of the dECM origin (Fig. 4B–D). Mechanistically, tissue fluidity is governed by signaling pathways that regulate cytoskeletal dynamics and cellular motility. Mitochondria provides energy and biosynthetic precursors required for cytoskeletal remodeling and adaptation to the dynamic extracellular matrix. In keeping with results presented above, VEGFR^WT^ Sk-Mel-31 cells showed alteration of the mitochondria network, with a balance shifted toward elongated structure, when adhered to dECM derived from VEGFR2^R1032Q^ SK-Mel-31 cells. Again, VEGFR2^R1032Q^ Sk-Mel-31 cells remained stable in their mitochondrial structure regardless of the dECM origin (Fig. 4E). These results confirmed the idea that ECM affected the behavior of tumor cells in tissues.

### ERK and PI3K signaling pathways mediate ECM remodeling in VEGFR2^R1032Q^ Sk-Mel-31 cells

**Fig. 5:**
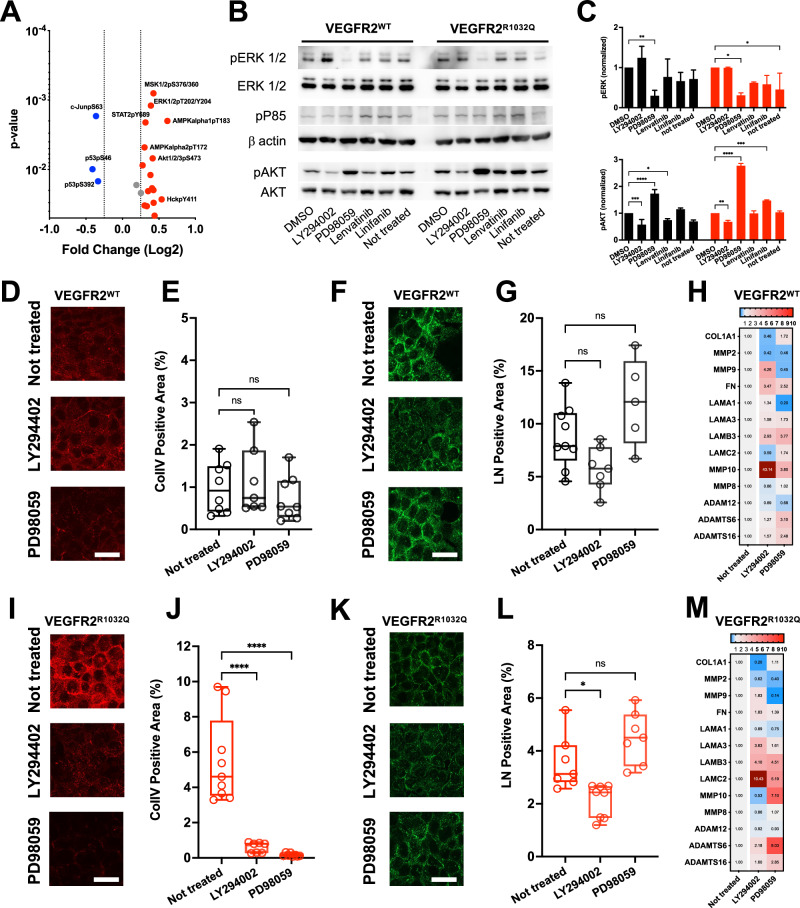
Signaling pathway modulation and ECM remodeling in VEGFR2^WT^ Sk-Mel-31 and VEGFR2^R1032Q^ Sk-Mel-31 cells. **A** Volcano plot of differentially phosphorylated proteins in VEGFR2^R1032Q^ Sk-Mel-31 cells vs VEGFR2^WT^ Sk-Mel-31 cells, upregulated (red) and downregulated (blue) signaling nodes. **B** Western blot analysis of ERK and AKT signaling pathways in VEGFR2^WT^Sk-Mel-31 and VEGFR2^R1032Q^ Sk-Mel-31 cell treated with LY294002, PD98059, Linifanib, and Lenvatinib. β-actin serves as a loading control. ERK1/2 (both total and phosphorylated) were analyzed from 5 µg of total lysate. P85, actin, and AKT (both total and phosphorylated) were analyzed from 20 µg of total lysate. **C** Quantification of pERK and pAKT levels, normalized to total ERK and AKT, respectively. Data are presented as mean ± SD from two independent experiments. Statistical significance was determined by two-way ANOVA, *P < 0.05, **P < 0.01, ***P < 0.001, ****P < 0.0001. **D** Representative images of collagen IV (ColIV) immunofluorescence staining in VEGFR2^WT^ Sk-Mel-31 under treatment conditions where indicated. Scale bar, 20 µm. **E** Quantification of ColIV-positive area. Single data are represented as empty dots in Min to Max box & whiskers plot (*n* = 7–8). Statistical significance was determined by one-way ANOVA. **F** Representative images of laminin (LN) immunofluorescence staining in VEGFR2^WT^Sk-Mel-31 under treatment conditions where indicated. Scale bar, 20 µm. **G** Quantification of LN-positive area. Single data are represented as empty dots in Min to Max box & whiskers plot (*n* = 5–9). Statistical significance was determined by one-way ANOVA, ****P < 0.0001. **H** Heatmap showing ECM-related gene expression in VEGFR2^WT^Sk-Mel-31 tumors under different conditions. **I** Representative images of collagen IV (ColIV) immunofluorescence staining in VEGFR2^R1032Q^ Sk-Mel-31 under treatment conditions where indicated. Scale bar, 20 µm. **J** Quantification of ColIV-positive area. Single data are represented as empty dots in Min to Max box & whiskers plot (*n* = 7–9). Statistical significance was determined by one-way ANOVA, ****P < 0.0001. **K** Representative images of laminin (LN) immunofluorescence staining in VEGFR2^R1032Q^ Sk-Mel-31 cells treated as indicated. Scale bar, 20 µm. **L** Quantification of LN-positive area. Single data are represented as empty dots in Min to Max box & whiskers plot (*n* = 7–9). Statistical significance was determined by one-way ANOVA, *P < 0.05. **M** Heatmap showing ECM-related gene expression in VEGFR2^R1032Q^ Sk-Mel-31 tumors under different conditions (*n* = 3–4).

To elucidate the potential signaling mechanisms underlying ECM remodeling in VEGFR2^R1032Q^ Sk-Mel-31 cells, we compared in vitro data of phospho-kinase array obtained from both confluent isogenic cell lysates. These analyses identified significant activation of ERK1/2, p85, and AKT1/2/3 pathways in VEGFR2^R1032Q^ Sk-Mel-31 cells (Fig. 5A and Supplementary Fig. 2A, B).

On the contrary, a marked reduction in p53 and c-Jun phosphorylation was observed in VEGFR2^R1032Q^ Sk-Mel-31 cells (Fig. 5A). Consistent with these findings, VEGFR2^R1032Q^ Sk-Mel-31 cells exhibited a significantly altered ECM profile compared to their VEGFR2^WT^ counterparts, with increased deposition of fibronectin, and collagen IV, while laminin and other ECM components were reduced (Supplementary Fig. 2C–G). To determine whether the ECM remodeling driven by the expression of VEGFR2^R1032Q^ is mediated via the PI3K and MAPK pathways, cells were treated with the PI3K inhibitor LY294002 and the MAPK inhibitor PD98059 and assessed for their effects on ECM gene expression and protein deposition. The efficacy of these treatments was confirmed by western blot analysis, showing significantly reduced phosphorylation of AKT at Ser473 (pS473) and pP85 following LY294002 treatment, and decreased ERK1/2 phosphorylation after PD98059 treatment (Fig. 5B, C). As expected, both Lenvatinib and Linifanib, two VEGFR2 inhibitors, prevented the activation of these signaling, confirming the role of VEGFR2 expression in their modulation [25, 26].

Interestingly, the treatment with LY294002 or PD98059 inhibitors reduced the expression of specific ECM components at both transcriptional and protein levels. Immunofluorescence analysis was employed to evaluate the effects of PI3K and MAPK pathway inhibitors on collagen IV and laminin deposition in Sk-Mel-31 cells. In VEGFR^WT^ Sk-Mel-31 cells, ECM deposition remained mostly unaffected by treatment with either LY294002 or PD98059, with no significant changes observed in collagen IV (Fig. 5D, E) or in laminin subunit alpha-1 (LAMA1) (Fig. 5F, G) levels across untreated, LY294002-treated, and PD98059-treated groups. Moreover, both treatments only partially modified the composition of the matrix (Fig. 5H). In contrast, VEGFR^R1032Q^ Sk-Mel-31 cells exhibited a marked reduction in ECM deposition following inhibitor treatments. Collagen IV deposition was significantly decreased after both LY294002 and PD98059 treatments (Fig. 5I, J). Laminin deposition was also markedly reduced in LY294002-treated VEGFR^R1032Q^ Sk-Mel-31 cells. However, no statistically significant reduction in laminin levels was observed in PD98059-treated VEGFR2^R1032Q^ Sk-Mel-31 cells (Fig. 5K, L). In keeping with these, both treatments markedly affect ECM remodeling (Fig. 5M).

### Lenvatinib inhibited VEGFR2^R1032Q^ -mediated ECM remodeling in vivo

**Fig. 6:**
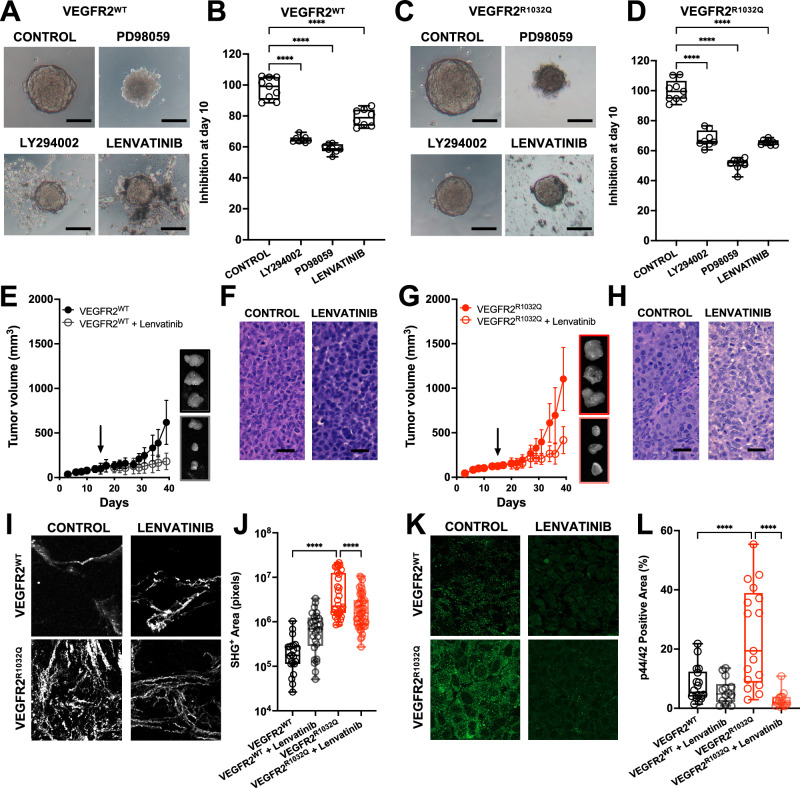
Lenvatinib treatment prevented ECM remodeling in VEGFR2^R1032Q^ Sk-Mel-31 derived tumors in vivo. **A** Representative images of 3D spheroids treated with PD98059, LY294002, or Lenvatinib at day 10 of VEGFR2^WT^Sk-Mel-31 cells. Scale bar 100 µm. **B** Quantification of inhibition at day 10. Single data are represented as empty dots in Min to Max box & whiskers plot (*n* = 12). Statistical significance was determined by two-way ANOVA, ****P < 0.0001. **C** Representative images of 3D spheroids treated with PD98059, LY294002, or Lenvatinib at day 10 of VEGFR2^R1032Q^ Sk-Mel-31 cells. Scale bar 100 µm. **D** Quantification of inhibition at day 10. Single data are represented as empty dots in Min to Max box & whiskers plot (*n* = 12). Statistical significance was determined by two-way ANOVA, ****P < 0.0001. **E** Tumor growth curves of VEGFR2^WT^ Sk-Mel-31-treated or not with Lenvatinib. Arrow indicates treatment start. Insets show representative ex vivo tumors at the endpoint. Data are presented as mean ± SD. **F** Representative H&E staining of FFPE slices from VEGFR2^WT^Sk-Mel-31-derived tumors treated or not with Lenvatinib. Scale bars, 50 µm. **G** Tumor growth curves of VEGFR2^R1032Q^ Sk-Mel-31 treated or not with Lenvatinib. Arrow indicates treatment start. Insets show representative ex vivo tumors at the endpoint. Data are presented as mean ± SD (*n* = 11–12). **H** Representative H&E staining of FFPE slices from VEGFR2^R1032Q^ Sk-Mel-31-derived tumors treated or not with Lenvatinib. Scale bars, 50 µm. **I** Representative second-harmonic generation (SHG) microscopy images showing collagen fiber organization in VEGFR2^WT^Sk-Mel-31 and VEGFR2^R1032Q^Sk-Mel-31-derived tumors treated or not with Lenvatinib. Scale bars, 50 µm. **J** Quantification of SHG-positive areas. Single data are represented as empty dots in Min to Max box & whiskers plot (*n* = 20–50). Statistical significance was determined by two-way ANOVA, ****P < 0.0001. **K** Representative immunofluorescence images of phosphorylated ERK (p44/42) Sk-Mel-31-derived tumors treated or not with Lenvatinib. Scale bars, 50 µm. **L** Quantification of p44/42-positive area. Single data are represented as empty dots in Min to Max box & whiskers plot (*n* = 20–25). Statistical significance was determined by two-way ANOVA, ****P < 0.0001.

To investigate whether VEGFR2 inhibitors mitigate ECM remodeling driven by VEGFR2^R1032Q^ expression, both cell lines were grown as 3D aggregates and treated with LY294002, PD98059, and Lenvatinib (Fig. 6A–D). Quantification of spheroid size at day 10 revealed a significant reduction in growth in VEGFR^R1032Q^ Sk-Mel-31 cells following Lenvatinib treatment (65% of control, Fig. 6D), with a less pronounced effect observed in VEGFR^WT^ Sk-Mel-31 spheroids (79% of control Fig. 6B). These findings were corroborated in vivo. Lenvatinib treatment reduced tumor growth in both Sk-Mel-31-derived tumors (Fig. 6E–H), with marked suppression of VEGFR^R1032Q^ Sk-Mel-31-derived tumors (Fig. 6G), stabilizing the architecture of the lesions (Fig. 6H). These observations were validated by SHG analysis (Fig. 6I). Lenvatinib treatment did not modify the collagen architecture in VEGFR^WT^ Sk-Mel-31-derived tumors. However, significantly reduced SHG signal intensity was observed in VEGFR^R1032Q^ Sk-Mel-31-derived tumors (Fig. 6I, J). Moreover, immunofluorescence analysis of phosphorylated ERK1/2 (p44/42 MAPK) showed diminished pathway activation in Lenvatinib-treated VEGFR2^R1032Q^ samples, correlating with impaired ECM remodeling (Fig. 6K, L). These findings highlight that Lenvatinib effectively attenuates VEGFR2^R1032Q^-driven ECM remodeling in vivo, primarily through inhibition of the ERK/MAPK pathway.

## Discussion

ECM is an active and dynamic component of the tumor microenvironment. The interstitial matrix forms porous three-dimensional networks around cells that interconnect cells in the stroma and actively shapes cancer progression through modulation of tissue stiffness, growth, factor bioavailability, immune cell infiltration, and cell behavior [27]. Here, we uncover a mechanism by which aberrant VEGFR2 activation drives extensive ECM remodeling. Beyond its classical role in the angiogenic switch, VEGFR2 sustains oncogenic programs through autocrine signaling that enhances tumor cell survival, invasion, and stemness. Notably, the substitutions R1051Q and D1052N constitutively activate VEGFR2 kinase activity [9], whereas R1032Q induces aberrant signaling via heterodimerization with the wild-type receptor [12]. Our data demonstrate that, in a melanoma model, expression of VEGFR2^R1032Q^ leads to increased collagen deposition, disorganized fibrillar networks, and transcriptional rewiring of ECM-modulating genes. Similar results were previously reported in the syngeneic Sk-Mel-31 melanoma model expressing constitutively active VEGFR2^R1051Q^ [28]. The impact of VEGFR2 hyperactivation is further confirmed in OVCAR3-derived ovarian cancer cells, where VEGFR2 is highly phosphorylated and its downregulation restores ECM organization. Importantly, in ovarian cancer, ECM remodeling and distinct ECM-related signatures correlate with poor prognosis [29]. Indeed, signaling factors released by omental preadipocytes, inflammatory, and stromal cells promote ECM remodeling by driving upregulation of collagens, matrix metalloproteinases, and other remodeling enzymes. Inhibition of insulin-like growth factor (IGF1) signaling, for example, reduces collagen deposition and tumor burden in subcutaneous and intraperitoneal ovarian cancer models [30].

While the role of the ECM in modulating tumor behavior is increasingly recognized, its regulation by specific growth factors remains to be fully understood. TGFβ drives cancer-associated fibrosis by modulating the expression of collagen 1A1 and 3A1, fibronectin and tenascin c via SMAD pathways [31, 32], controlling collagen linearization through the induction of WISP1, and of lysyl oxidase and metalloproteases [33]. Platelet derived growth factor (PDGF) remodels tumor ECM through the activation of the cancer associated fibroblast [34]. In contrast, VEGF/VEGFR2 axis contribution to ECM dynamics in solid tumors is largely unexplored. Our findings reveal that loss of fine VEGFR2 regulation in cancer cells profoundly alters ECM composition and structure, promoting tumor progression. Of note, the ECM structure of VEGFR2^R1032Q^ -Sk-Mel-31-derived tumors mirrors the architecture observed in human melanoma samples derived by patients harboring VEGFR2^R1032Q^ (TCGA-FW-A3R5; TCGA-FR-A7U9; TCGA-EE-A2MD; available on cBioportal 8/11/2025) [35]. These lesions are characterized by dense, widely distributed ECM. While alternative pathways cannot be excluded, aberrant VEGFR2 likely contributes to this phenotype. The resulting chaotic collagen network supports cancer cell activation and induces a transition from solid to fluid state of tissue. Tumors, traditionally considered rigid, space-occupying-solid mass, can acquire soft behavior [36, 37]. In a tumor, malignant cells are not just a solidified lumped mass but are embedded into a fibrotic matrix. Evidence suggests that tumor softening involves a rigid backbone of non-motile, jammed cells that physically support the mass, interspersed with fluid regions of unjammed, motile cells capable of escaping the tumor [38, 39].

The ECM remodeling not only provides mechanical cues but also may act as a barrier that impedes the effective penetration and distribution of therapeutic agents. Preclinical models have demonstrated that irregular collagen architecture can hinder the infiltration by T cells and other effector cells, thereby fostering immunosuppressive niches within the tumor microenvironment [40]. In colorectal cancer, for example, desmoplastic tumors characterized by dense ECM deposition, reduced expression of PROX1, and altered collagen crosslinking show increased resistance to chemotherapy, and exhibit features of immune exclusion and aberrant angiogenesis. Similar immune-silent and hyper vascular phenotypes have been observed in colon rectal carcinoma models harboring VEGFR2 mutation, where enhanced ECM remodeling contributes to immune-evasive microenvironments. However, the role of ECM remodel in immunological activation is still controversial. Several studies demonstrate that the bioactive ECM fragments, known as matrikines, modulate immune response either positively or negatively, further illustrating the complex and context-dependent interplay between ECM and immunity [41, 42].

We can conclude that a fine characterization of ECM remodeling and its regulators may enhance immunotherapy and chemotherapy by restoring immune cell infiltration. Strategies to normalize the ECM, including inhibition of hyaluronidase, lysyl oxidase, LOXL2, and matrix metalloproteinases, are under investigation. Although early trials were limited by low efficacy or toxicity, improved understanding of their context-dependent regulation has reinvigorated interest [43, 44]. For instance, in hepatocellular carcinoma, the EGFR inhibitor Erlotinib reduced liver fibrosis and prevented malignant transformation in pre-neoplastic lesions with minimal effects on cancer cells [45].

Collectively, our results demonstrate that aberrant VEGFR2 phosphorylation leads to remodel ECM, potentially contributing to disease progression by affecting both structural protein deposition and transcriptional regulation. Notably, these ECM alterations can be attenuated or partially reversed not only by pathway-specific inhibitors targeting ERK and PI3K, but also by multi-kinase inhibitors such as lenvatinib. Thus, the so-called “field effect” therapy whereby targeting ECM-related signaling pathways may be combined with chemo- and immunotherapy to enhance treatment efficacy in solid tumors.

## Materials and methods

### Cell cultures

Human melanoma Sk-Mel-31 cells, provided by Memorial Sloan Kettering Cancer Center (MSK00005207), were grown in RPMI (Thermo Fisher Scientific, Carlsbad, CA, USA) supplemented with 10% Fetal Bovine Serum (FCS) (Thermo Fisher Scientific), 1% non-essential amino acids (NEAA) (Thermo Fisher Scientific) and 1% Pen/Strep (Thermo Fisher Scientific). Sk-Mel-31 stably expresses VEGFR2^WT^ and VEGFR2^R1032Q^ were generated using polyethylenimine (PEI) (Sigma-Aldrich, St. Louis, MO, USA) transfection reagent and selected by 0.5 mg/mL geneticin. OVCAR3 was purchased from the European Collection of Authenticated Cell Cultures (ECACC). OVCAR3 cells were maintained in RPMI medium (Thermo Fisher Scientific) supplemented with 20% FBS, and 0.01 mg/mL bovine insulin. For VEGFR2 silencing, cells were infected with lentiviral particles harboring Mission® VEGFR2-targeting shRNAs as published in Grillo et al. [9]. Cells transduced with non-targeting lentiviral particles (SCH202V) were used as controls in all experiments. Transduced cells were maintained in 1 μg/mL puromycin. Cells were tested monthly for mycoplasma contamination.

### Mutagenesis

pBE_hVEGFR2 plasmid encoding for wild-type hVEGFR2 [NM_002253.2] was kindly provided by Prof. Kurt Ballmer-Hofer (Paul Scherrer Institut, PSI, Villigen, Switzerland). pBE_hVEGFR^R1032Q^ plasmid was generated using the QuikChange Lightning Site-directed Mutagenesis Kit (Agilent Technologies, Santa Clara, CA, USA). To introduce R1032Q point mutation the following primers were used: forward 5’-GGGACCTGGCGGCACAAAATATCCTCTTATCG-3’; reverse 5’-CGATAAGAGGATATTTTGTGCCGCCAGGTCCC-3’.

### Mouse tumor generation

In total, 4 × 10^6^ VEGFR2^WT^ Sk-Mel-31 or VEGFR2^R1032Q^ Sk-Mel-31 cells were subcutaneously injected (s.c.) into the flanks of 6 weeks old NOD/SCID mice (Envigo, Udine, Italy). All animals were purchased at the same age, with males and females represented in similar proportions. at the same age, with males and females represented in similar proportions. Tumor volumes were measured using calipers and calculated according to the formula V = (D × d^2^)/2, where D and d are the major and minor perpendicular tumor diameters, respectively. Lenvatinib (Sigma-Aldrich) was administered at 20 mg/kg in 8% ethanol by oral gavage every day, after mice randomization once tumor volume reached 100 mm^3^. At the endpoint of the experimental procedure, tumors were harvested, weighed, and processed for downstream analyses. Animals were randomly allocated to experimental groups using a random number generator to ensure unbiased distribution. No blinding analysis was performed. In accordance with our institutional guidelines, tumor size did not exceed 1000 mm^3^ in volume or 2 g. All in vivo experiments were approved by the local animal ethics committee (OPBA, Organismo Preposto al Benessere degli Animali, Università degli Studi di Brescia, Brescia, Italy) and Italian “Ministero della Salute” (Authorization n. 266/2016) and conducted in accordance with national guidelines.

### Histological staining

Formalin-fixed paraffin-embedded (FFPE) tissue sections were stained with hematoxylin and eosin (H&E), Masson’s trichrome, and Periodic Schiff acid. Nuclei were stained with 4’,6-diamidino-2-phenylindole (DAPI) (Sigma-Aldrich) or TO-PRO-3 (Molecular Probes, Eugene, OR, USA). Images were captured using LSM880 confocal microscope (Carl Zeiss, Oberkochen, Germany) and analyzed by ImageJ software. For Second Harmonic Generation (SHG), collagen bundles were collected using LSM880 two-photon microscope associated with Chameleon IR Laser (Ex λ = 860, Em λ = 430/10) equipped with a Plan-Neofluar 20×/0.5 NA and 40×/0.75 objective (Carl Zeiss).

### Microarray data processing and analysis

To profile gene expression, total RNA was converted to cRNA subsequently into cDNA and hybridized, onto a Human Clariom S GeneChip (Thermo Fisher Scientific) following the manufacturer’s guidelines. Gene-level expression of over 20,000 well-annotated genes was assessed. A 3000 7 G Scanner (Thermo Fisher Scientific) was used in conjunction with GeneChip Operation Software (Thermo Fisher Scientific) to generate a single CEL file for each hybridized cDNA. Filtered datasets were further processed for pathway analysis and gene set enrichment using Gene Set Enrichment Analysis (GSEA) and Gene Ontology (GO) gene-set lists.

### Gene expression

Total RNA was extracted using TRIzol Reagent (Thermo Fisher Scientific) according to the manufacturer’s instructions. Complementary DNA (cDNA) was made from 2 μg of total RNA using MML-V (Thermo Fisher Scientific). Real-time PCR was performed using ViiA7 Real-Time PCR System (Thermo Fisher Scientific), and data was analyzed with Viia7 Real-Time Software (Thermo Fisher Scientific).

The oligonucleotide primers used are listed in Table 1.

**Table 1:** List of human primers used in this research.

Gene name	Forward primer	Reverse primer
*Hs_LAMA1*	GGCCCTGTGTTTGTAAGGAA	TCTTGCTGAGACGGGATCTT
*Hs_LAMA3*	TTGATCCGCTGGCATTTCCT	CAGCACCCCAGGATGGATAA
*Hs_LAMB3*	AGCTTTCAGGCGATCTGGAG	CCCAACAGGTGGATAGCAGG
*Hs_LAMC2*	CAAAGGTTCTCTTAGTGCTCGAT	CACTTGGAGTCTAGCAGTCTCT
*Hs_COL1A1*	AAGAGGAAGGCCAAGTCGAG	AGATCACGTCATCGCACAAC
*Hs_COL1A2*	TTTAATTTTTCTGCTTGCCCA	CAAAACACTTTCCCATGAGTG
*Hs_FN*	TATGTGGTCGGAGAAACGTG	TCCTTGTGTCCTGATCGTTG
*Hs_EDB*	CCACCATTATTGGGTACCGC	CGCATGGTGTCTGGACCAATG
*Hs_MMP2*	GTATGGCTTCTGCCCTGAGA	CACACCACATCTTTCCGTCA
*Hs_MMP8*	CATGTTCTCCCTGAAGACGCT	ACTTTTCCAGGTAGTCCTGAAC
*Hs_MMP9*	GCCACTTCCCCTTCATCTTC	GCCGTCCTGGGTGTAGAGT
*Hs_MMP10*	GACAGAAGATGCATCAGGCAC	GGCCCTGTGTTTGTAAGGAA
*Hs_ADAM12*	ACACGGTAATTCTGGGTCACT	ACACGTGCTGAGACTGACTG
*Hs_ADAMTS6*	GTGATCCTGACAGTAAGCCACC	CCACCATCACAAGTCTTGCTGC
*Hs_ADAMTS16*	ATAGGAGTCGCCTCTGCACCAA	AGCACGGAAGTCAACACTGTCC

### Immunofluorescence

VEGFR2^WT^ Sk-Mel-31 and VEGFR2^R1032Q^ Sk-Mel-31 were grown for 10 days in complete medium, fixed with PFA 4% in PBS for 20 min at 4 °C, and maintained in PBS 2% BSA. Then, samples were incubated with anti-CollIV antibody (HEYL, Germany) or with anti-Laminin 1 (L9393 -Sigma-Aldrich) followed by Alexa Fluor 594 anti-rabbit IgG (A-11012 -Molecular Probes) or Alexa Fluor 488 anti-mouse IgG (A11028-Molecular Probes). Nuclei were counterstained with TO-PRO-3 iodide (T3605-Molecular Probes). Formalin-fixed paraffin-embedded (FFPE) tissue sections were stained with anti-phospho-p44/24 MAPK antibody (pThr202/Thr204, #9101- Cell Signaling Technology, Danvers, MA, USA) antibody or pVEGFR2 (#2478.-Cell Signaling Technology) when indicated. Antigen retrieval was performed in Citrate Buffer solution (0.1 M and pH 6.0) at 95 °C for 20 min before immunostaining. Nuclei were counterstained with TO-PRO-3 iodide (Molecular Probes). Images were captured using Axio Observer (Carl Zeiss) equipped Apotome.2 and with Plan-Apochromat 63×/1.4 Oil DIC objective (Carl Zeiss) and analyzed by Zen software (Carl Zeiss).

### Sk-Mel-31 spheroid

In all, 2000 cells aggregated in spheroids in 20% methylcellulose RPMI medium, were plated in a low attachment round-bottom 96-well plate for 48 h. On day 3, spheroids were transferred in a 24-well and treated with 4 µM LY294002 (Sigma-Aldrich), 4 µM PD98059 (Sigma-Aldrich), and 40 µM Lenvatinib (Sigma-Aldrich) every 2 days. Spheroids were photographed every 24 h using an Primovert microscope equipped with a Plan-Apochromat 10X/0,25PH1 objective (Carl Zeiss).

### Matrix decellularization

In total, 3 × 10^5^ cells/cm^2^ Sk-Mel-31 VEGFR2^WT^ and VEGFR2^R1032Q^ were seeded in complete medium and grown for eight days. Decellularized matrices were obtained with gently washed monolayers in PBS/EDTA 4 mM until the complete removal of seeded cells.

### Tissue fluidity

Tissue fluidity was assessed by time-lapse video microscopy. Cells were seeded at 100,000 cells/cm^2^ in 24-well plates overnight. Cells were observed using Axio Observer (Zeiss Axiovert), and phase-contrast snap photographs (1 frame every 10 min) were digitally recorded for 1200 min. Constant temperature (37 °C) and pCO2 (5%) were maintained throughout the experimental period by means of a heatable stage and climate chamber. The videos obtained were analyzed using particle image velocimetry (PIV) with the MATLAB application PIVlab (https://it.mathworks.com/matlabcentral/fileexchange/27659-pivlab-particle-image-velocimetry-piv-tool-with-gui) to compute the velocity vectors of each frame [46]. The velocity vectors were generated with an interrogation area of Pass1 100 × 100 px [56 µm^2^] and Pass 2 of 50 × 50 px [28 µm^2^]. Mean and standard deviation were determined from two independent experiments.

### Mitochondria network

Cells were seeded at 100,000 cells/cm^2^ in eight-well into ibidi plates overnight and stained with MitoTracker (Thermo Fisher Scientific). Images were captured using LSM880 confocal microscope (Carl Zeiss) and analyzed by ImageJ software.

### Western blot

Cells were lysed in lysis buffer [50 mM Tris-HCl buffer (pH 7.4), 150 mM NaCl, 1% Triton X-100, 1 mM Na_3_VO_4_, and protease and phosphatase inhibitors (Sigma-Aldrich)]. Next, total proteins were separated by SDS-PAGE and probed with anti-phospho-AKT antibody (pSer473, #4060- Cell Signaling Technology), anti-phospho-p44/24 MAPK antibody (pThr202/Thr204, #4370-Cell Signaling Technology), anti-phospho-p85 (pTyr458, #4228-Cell Signaling Technology). Phospho-protein levels were normalized using anti-p44/42 MAPK (#9102- Cell Signaling Technology), anti-b-actin Clone AC-74 (A2228-Sigma-Aldrich), or anti-AKT (#4691-Cell Signaling Technology) antibodies. The chemiluminescent signal was acquired by ChemiDoc^TM^ Imaging System (BioRad, Hercules, CA, USA) and analyzed with ImageLab software from BioRad.

### Statistical analyses

Statistical analyses were performed using the statistical package Prism10 (GraphPad Software, San Diego, CA, USA). Two-tailed Student *t* test or ordinary one-way ANOVA or two-way ANOVA were used to determine statistical significance. *P < 0.05, **P < 0.01, ***P < 0.001, ****P < 0.0001. Error bars denote ± SD.

## Supplementary information

checklist

Supplementary material

Supplementary table 1

## References

[1] Frantz C, Stewart KM, Weaver VM. The extracellular matrix at a glance. J Cell Sci. 2010;123:4195–200. 21123617 10.1242/jcs.023820 PMC2995612

[2] Saraswathibhatla A, Indana D, Chaudhuri O. Cell-extracellular matrix mechanotransduction in 3D. Nat Rev Mol Cell Biol. 2023;24:495–516. 36849594 10.1038/s41580-023-00583-1 PMC10656994

[3] Vasudevan J, Jiang K, Fernandez JG, Lim CT. Extracellular matrix mechanobiology in cancer cell migration. Acta Biomater. 2023;163:351–64. 36243367 10.1016/j.actbio.2022.10.016

[4] Hu Q, Wang Y, Yao S, Mao Y, Liu L, Li Z, et al. Desmoplastic reaction associates with prognosis and adjuvant chemotherapy response in colorectal cancer: a multicenter retrospective study. Cancer Res Commun. 2023;3:1057–66. 37377615 10.1158/2767-9764.CRC-23-0073 PMC10269709

[5] Wolf B, Weydandt L, Dornhöfer N, Hiller GGR, Höhn AK, Nel I, et al. Desmoplasia in cervical cancer is associated with a more aggressive tumor phenotype. Sci Rep. 2023;13:18946. 37919378 10.1038/s41598-023-46340-4 PMC10622496

[6] Lo A, Wang LS, Scholler J, Monslow J, Avery D, Newick K, et al. Tumor-promoting desmoplasia is disrupted by depleting FAP-expressing stromal cells. Cancer Res. 2015;75:2800–10. 25979873 10.1158/0008-5472.CAN-14-3041 PMC4506263

[7] Guo M, Zhang J, Han J, Hu Y, Ni H, Yuan J, et al. VEGFR2 blockade inhibits glioblastoma cell proliferation by enhancing mitochondrial biogenesis. J Transl Med. 2024;22:419. 38702818 10.1186/s12967-024-05155-1 PMC11067099

[8] Toledo RA, Garralda E, Mitsi M, Pons T, Monsech J, Vega E, et al. Exome sequencing of plasma DNA portrays the mutation landscape of colorectal cancer and discovers mutated VEGFR2 receptors as modulators of antiangiogenic therapies. Clin Cancer Res. 2018;24:3550–9. 29588308 10.1158/1078-0432.CCR-18-0103

[9] Grillo E, Corsini M, Ravelli C, di Somma M, Zammataro L, Monti E, et al. A novel variant of VEGFR2 identified by a pan-cancer screening of recurrent somatic mutations in the catalytic domain of tyrosine kinase receptors enhances tumor growth and metastasis. Cancer Lett. 2021;496:84–92. 33035615 10.1016/j.canlet.2020.09.027

[10] Paley PJ, Staskus KA, Gebhard K, Mohanraj D, Twiggs LB, Carson LF, et al. Vascular endothelial growth factor expression in early stage ovarian carcinoma. Cancer. 1997;80:98–106. 9210714 10.1002/(sici)1097-0142(19970701)80:1<98::aid-cncr13>3.0.co;2-a

[11] Yamamoto S, Konishi I, Mandai M, Kuroda H, Komatsu T, Nanbu K, et al. Expression of vascular endothelial growth factor (VEGF) in epithelial ovarian neoplasms: correlation with clinicopathology and patient survival, and analysis of serum VEGF levels. Br J Cancer. 1997;76:1221–7. 9365173 10.1038/bjc.1997.537 PMC2228134

[12] Ravelli C, Corsini M, Bresciani R, Rizzo AM, Zammataro L, Corsetto PA, et al. Cancer-associated VEGFR2. Neoplasia. 2025;67:101195. 40517532 10.1016/j.neo.2025.101195 PMC12206127

[13] Mosquera-Zamudio A, Pérez-Debén S, Porcar-Saura S, Casabó-Vallés G, Martínez-Rodríguez M, Garzón MJ, et al. Beyond nest size: the clinicopathological spectrum of large nested melanocytic tumours and the value of comparative genomic hybridisation and messenger RNA expression analysis. Pathology. 2025;57:40–8. 39489642 10.1016/j.pathol.2024.08.002

[14] Püspöki Z, Storath M, Sage D, Unser M. Transforms and operators for directional bioimage analysis: a survey. Adv Anat Embryol Cell Biol. 2016;219:69–93. 27207363 10.1007/978-3-319-28549-8_3

[15] Liu Z, Pouli D, Sood D, Sundarakrishnan A, Hui Mingalone CK, Arendt LM, et al. Automated quantification of three-dimensional organization of fiber-like structures in biological tissues. Biomaterials. 2017;116:34–47. 27914265 10.1016/j.biomaterials.2016.11.041 PMC5210183

[16] Bacchetti R, Yuan S, Rainero E. ADAMTS proteases: their multifaceted role in the regulation of cancer metastasis. Dis Res. 2024;4:40–52. 38948119 10.54457/DR.202401004 PMC7616120

[17] Pignata P, Apicella I, Cicatiello V, Puglisi C, Magliacane Trotta S, Sanges R. et al. Prolyl 3-hydroxylase 2 is a molecular player of angiogenesis. Int J Mol Sci. 2021;22:3896 33918807 10.3390/ijms22083896 PMC8069486

[18] Roshna Sankar SJ, Sharma K, Devi P, Chundawat P, Shalini G. Shalini Gupta,. Integrin α11: key signaling pathways and tumor dynamics. J Oral Maxillofac Surg Med Pathol. 2025;37:540–551.

[19] Heljasvaara R, Aikio M, Ruotsalainen H, Pihlajaniemi T. Collagen XVIII in tissue homeostasis and dysregulation—lessons learned from model organisms and human patients. Matrix Biol. 2017;57-58:55–75. 27746220 10.1016/j.matbio.2016.10.002

[20] Walker C, Mojares E, Del R¡o Hern ndez A. Role of extracellular matrix in development and cancer progression. Int J Mol Sci. 2018;19:3028 30287763 10.3390/ijms19103028 PMC6213383

[21] Huang Y, Xu X, Lu Y, Sun Q, Zhang L, Shao J, et al. The phase separation of extracellular matrix protein matrilin-3 from cancer-associated fibroblasts contributes to gastric cancer invasion. FASEB J. 2024;38:e23406. 38193601 10.1096/fj.202301524R

[22] Rocco D, Tortora A, Marotta V, Machado AM, Selistre-de-Araújo HS, Vitale M. Integrin-fibronectin interaction is a pivotal biological and clinical determinant in papillary thyroid carcinoma. Endocr Relat Cancer 2025;32:6. 10.1530/ERC-25-0101 PMC12150248 40423510

[23] Kveiborg M, Albrechtsen R, Couchman JR, Wewer UM. Cellular roles of ADAM12 in health and disease. Int J Biochem Cell Biol. 2008;40:1685–702. 18342566 10.1016/j.biocel.2008.01.025

[24] Massagué J, Sheppard D. TGF-β signaling in health and disease. Cell. 2023;186:4007–37. 37714133 10.1016/j.cell.2023.07.036 PMC10772989

[25] Luo Y, Jiang F, Cole TB, Hradil VP, Reuter D, Chakravartty A, et al. A novel multi-targeted tyrosine kinase inhibitor, linifanib (ABT-869), produces functional and structural changes in tumor vasculature in an orthotopic rat glioma model. Cancer Chemother Pharm. 2012;69:911–21. 10.1007/s00280-011-1740-7 22080168

[26] Mei Z, Gao X, Pan C, Wu Q, Wang S, Qian J, et al. Lenvatinib enhances antitumor immunity by promoting the infiltration of TCF1. Cancer Sci. 2023;114:1284–96. 36609997 10.1111/cas.15719 PMC10067412

[27] Naba A, Clauser KR, Ding H, Whittaker CA, Carr SA, Hynes RO. The extracellular matrix: tools and insights for the “omics” era. Matrix Biol. 2016;49:10–24. 26163349 10.1016/j.matbio.2015.06.003 PMC5013529

[28] Grillo E, Corsini M, Ravelli C, Zammataro L, Bacci M, Morandi A, et al. Expression of activated VEGFR2 by R1051Q mutation alters the energy metabolism of Sk-Mel-31 melanoma cells by increasing glutamine dependence. Cancer Lett. 2021;507:80–8. 33744390 10.1016/j.canlet.2021.03.007

[29] Olalekan S, Xie B, Back R, Eckart H, Basu A. Characterizing the tumor microenvironment of metastatic ovarian cancer by single-cell transcriptomics. Cell Rep. 2021;35:109165. 34038734 10.1016/j.celrep.2021.109165

[30] Waters JA, Robinson M, Lujano-Olazaba O, Lucht C, Gilbert SF, House CD. Omental preadipocytes stimulate matrix remodeling and IGF signaling to support ovarian cancer metastasis. Cancer Res. 2024;84:2073–89. 38635891 10.1158/0008-5472.CAN-23-2613 PMC11217736

[31] Mohammadi H, Sahai E. Mechanisms and impact of altered tumour mechanics. Nat Cell Biol. 2018;20:766–74. 29950570 10.1038/s41556-018-0131-2

[32] Meng C, He Y, Wei Z, Lu Y, Du F, Ou G, et al. MRTF-A mediates the activation of COL1A1 expression stimulated by multiple signaling pathways in human breast cancer cells. Biomed Pharmacother. 2018;104:718–28. 29807221 10.1016/j.biopha.2018.05.092

[33] Jia H, Janjanam J, Wu SC, Wang R, Pano G, Celestine M, et al. The tumor cell-secreted matricellular protein WISP1 drives pro-metastatic collagen linearization. EMBO J. 2019;38:e101302. 31294477 10.15252/embj.2018101302 PMC6694215

[34] Borkham-Kamphorst E, Alexi P, Tihaa L, Haas U, Weiskirchen R. Platelet-derived growth factor-D modulates extracellular matrix homeostasis and remodeling through TIMP-1 induction and attenuation of MMP-2 and MMP-9 gelatinase activities. Biochem Biophys Res Commun. 2015;457:307–13. 25576870 10.1016/j.bbrc.2014.12.106

[35] de Bruijn I, Kundra R, Mastrogiacomo B, Tran TN, Sikina L, Mazor T, et al. Analysis and visualization of longitudinal genomic and clinical data from the AACR Project GENIE Biopharma Collaborative in cBioPortal. Cancer Res. 2023;83:3861–7. 37668528 10.1158/0008-5472.CAN-23-0816 PMC10690089

[36] Murphy MC, Huston J, Glaser KJ, Manduca A, Meyer FB, Lanzino G, et al. Preoperative assessment of meningioma stiffness using magnetic resonance elastography. J Neurosurg. 2013;118:643–8. 23082888 10.3171/2012.9.JNS12519 PMC3920576

[37] Jamin Y, Boult JKR, Li J, Popov S, Garteiser P, Ulloa JL, et al. Exploring the biomechanical properties of brain malignancies and their pathologic determinants in vivo with magnetic resonance elastography. Cancer Res. 2015;75:1216–24. 25672978 10.1158/0008-5472.CAN-14-1997 PMC4384983

[38] Plodinec M, Loparic M, Monnier CA, Obermann EC, Zanetti-Dallenbach R, Oertle P, et al. The nanomechanical signature of breast cancer. Nat Nanotechnol. 2012;7:757–65. 23085644 10.1038/nnano.2012.167

[39] Guck J, Schinkinger S, Lincoln B, Wottawah F, Ebert S, Romeyke M, et al. Optical deformability as an inherent cell marker for testing malignant transformation and metastatic competence. Biophys J. 2005;88:3689–98. 15722433 10.1529/biophysj.104.045476 PMC1305515

[40] Sapudom J, Alatoom A, Tipay PS, Teo JC. Matrix stiffening from collagen fibril density and alignment modulates YAP-mediated T-cell immune suppression. Biomaterials. 2025;315:122900. 39461060 10.1016/j.biomaterials.2024.122900

[41] Lu X, Gou Z, Chen H, Li L, Chen F, Bao C, et al. Extracellular matrix cancer-associated fibroblasts promote stromal fibrosis and immune exclusion in triple-negative breast cancer. J Pathol. 2025;265:385–99. 39846260 10.1002/path.6395

[42] O’Connell BC, Hubbard C, Zizlsperger N, Fitzgerald D, Kutok JL, Varner J, et al. Eganelisib combined with immune checkpoint inhibitor therapy and chemotherapy in frontline metastatic triple-negative breast cancer triggers macrophage reprogramming, immune activation and extracellular matrix reorganization in the tumor microenvironment. J Immunother Cancer. 2024;12:e009160. 10.1136/jitc-2024-009160 PMC11367338 39214650

[43] Fields GB. The rebirth of matrix metalloproteinase inhibitors: moving beyond the dogma. Cells. 2019;8:984 31461880 10.3390/cells8090984 PMC6769477

[44] Huang J, Zhang L, Wan D, Zhou L, Zheng S, Lin S, et al. Extracellular matrix and its therapeutic potential for cancer treatment. Signal Transduct Target Ther. 2021;6:153. 33888679 10.1038/s41392-021-00544-0 PMC8062524

[45] Fuchs BC, Hoshida Y, Fujii T, Wei L, Yamada S, Lauwers GY, et al. Epidermal growth factor receptor inhibition attenuates liver fibrosis and development of hepatocellular carcinoma. Hepatology. 2014;59:1577–90. 24677197 10.1002/hep.26898 PMC4086837

[46] Jipp M, Wagner BD, Egbringhoff L, Teichmann A, Rübeling A, Nieschwitz P, et al. Cell-substrate distance fluctuations of confluent cells enable fast and coherent collective migration. Cell Rep. 2024;43:114553. 39150846 10.1016/j.celrep.2024.114553

