# Supplementary material for: Aberrant VEGFR2 supports tumor growth by extracellular matrix remodeling
Source: Cell Death Dis. 2026 Jan 15;17(1):169. doi: 10.1038/s41419-025-08404-3 (PMC12876860; doi:10.1038/s41419-025-08404-3)
Supplement: Supplementary file 2 — Supplementary material [file 41419_2025_8404_MOESM2_ESM.pdf]

Supplementary material

Title

Aberrant VEGFR2 supports tumor growth by extracellular matrix remodeling

Running title

VEGFR2-dependent ECM remodeling

Authors

Michela Corsini[1,2,*], Cosetta Ravelli[1,2], Mattia Domenichini[1], Anna Ventura[1], Camilla Maggi[1], Elisa Moreschi[1,2], Mirko Tamma[3], Chiara Romani[4,5], Claudia Piccoli[3], Elisabetta Grillo[1,2], and Stefania Mitola[1,2,*].

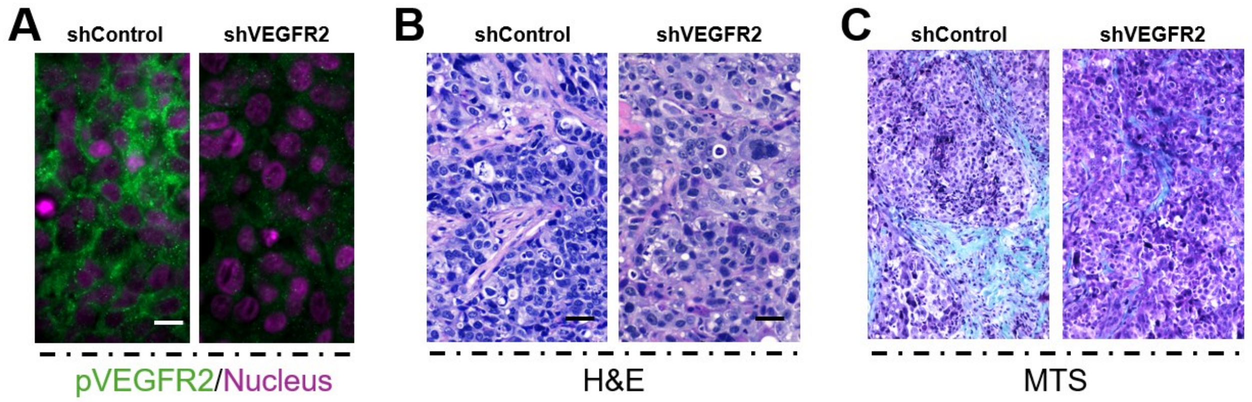

Supplementary Figure 1: VEGFR2 silencing reduces phosphorylation and ECM deposition (A) Representative pVEGFR2 staining of FFPE slices from shControl and shVEGFR2 OVCAR-derived tumors. pVEGFR2 signal in green, nuclear staining in magenta (n=4-5). Scale bars, 20 µm. (B-C) Representative H&E and MTS staining of FFPE slices from shControl and shVEGFR2 OVCAR-derived tumors. Scale bars, 50 µm. (n=4-5).

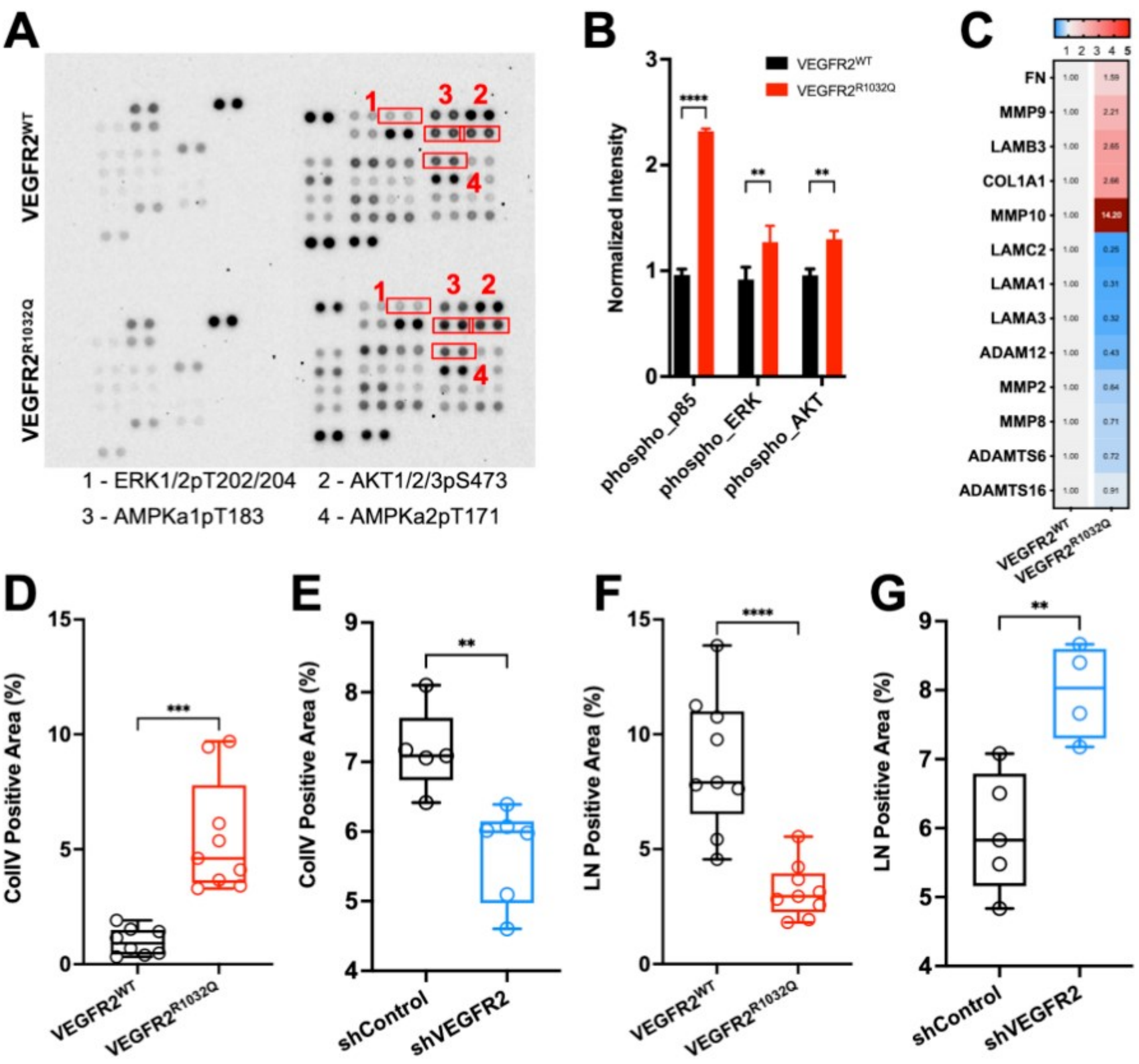

Supplementary Figure 2: Altered Signaling Pathway Modulation and ECM deposition in Sk-Mel-31-VEGFR2^{WT} and Sk-Mel-31-VEGFR2^{R1032Q} cells.

(A) Phospho-protein array of VEGFR2^{R1032Q} Sk-Mel-31 and VEGFR2^{WT} Sk-Mel-31 cells. Differentially phosphorylated proteins are highlighted in red. (B) Spot Quantification of pP85, pERK, and pAKT normalized to total protein levels. Data are presented as mean ± SD from two independent experiments. Statistical significance was determined by two-way ANOVA, $^{**}P < 0.01$, $^{****}P < 0.0001$.(C) Heatmap showing ECM-related gene expression in VEGFR2^{R1032Q} Sk-Mel-31 vs VEGFR2^{WT} Sk-Mel-31. (D) Quantification of ColIV-positive areas in VEGFR2^{R1032Q} Sk-Mel-31 and VEGFR2^{WT} Sk-Mel-31 cells. Single data are represented as empty dots in Min to Max box &

whiskers plot (n=7-9). Statistical significance was determined by two-way ANOVA, ***$P < 0.001$. (E) Quantification of ColIV-positive areas in shControl OVCAR3 and shVEGFR2 OVCAR cells. Single data are represented as empty dots in Min to Max box & whiskers plot (n=5-6). Statistical significance was determined by two-way ANOVA, **$P < 0.01$. (F) Quantification of LN-positive areas in $VEGFR2^{R1032Q}$ Sk-Mel-31 and $VEGFR2^{WT}$ Sk-Mel-31 cells. Single data are represented as empty dots in Min to Max box & whiskers plot (n=7-9). Statistical significance was determined by two-way ANOVA, ****$P < 0.0001$. (G) Quantification of LN-positive areas in shControl OVCAR3 and shVEGFR2 OVCAR cells. Single data are represented as empty dots in Min to Max box & whiskers plot (n=5-6). Statistical significance was determined by two-way ANOVA, **$P < 0.01$.
