# Supplementary material for: Aberrant VEGFR2 supports tumor growth by extracellular matrix remodeling
Source: Cell Death Dis. 2026 Jan 15;17(1):169. doi: 10.1038/s41419-025-08404-3 (PMC12876860; doi:10.1038/s41419-025-08404-3)
Supplement: Supplementary file 3 — Supplementary table 1 [file 41419_2025_8404_MOESM3_ESM.pdf]

ID	R1032Q Avg (log2)	WT Avg (log2)	Fold Change	P-val	FDR P-val	Gene Symbol
TC0100010923.hg.1	8,29	10,86	-5,91	3,64E-06	0,026	PRG4
TC0200015678.hg.1	10,32	12,62	-4,91	0,0003	0,2037	IGFBP5
TC1900009970.hg.1	4,93	7,22	-4,87	0,0028	0,5742	BST2
TC1000010870.hg.1	4,58	6,64	-4,18	0,0053	0,6757	SRGN
TC1600009580.hg.1	9,59	11,57	-3,94	0,0022	0,5304	XYLT1
TC1200006996.hg.1	6,9	8,84	-3,85	5,85E-05	0,1432	PIK3C2G
TC1600008005.hg.1	7,62	9,52	-3,74	6,52E-06	0,0349	ADGRG1
TC2200008036.hg.1	7,13	8,97	-3,59	0,0016	0,491	USP41
TC0900011300.hg.1	6,22	7,9	-3,2	0,012	0,7253	TNFSF15
TC2200006540.hg.1	7,75	9,4	-3,13	0,0001	0,1561	USP18
TC0X00011413.hg.1	8,48	10,12	-3,13	0,0052	0,6757	L1CAM
TC0400007344.hg.1	8,46	10,1	-3,11	6,01E-05	0,1432	UCHL1
TC0200007205.hg.1	5,85	7,39	-2,91	0,0036	0,6181	RASGRP3
TC2100007208.hg.1	9,89	11,43	-2,91	0,0104	0,7253	MX1
TC0300009727.hg.1	6,06	7,57	-2,86	0,0007	0,3684	VPS8
TC0X00010375.hg.1	5,98	7,47	-2,83	0,0005	0,321	ZMAT1
TC1000008400.hg.1	9,97	11,45	-2,8	0,0027	0,5723	IFIT1
TC0400012437.hg.1	13,38	14,86	-2,78	2,64E-05	0,1105	SCRG1
TC0200007200.hg.1	10,8	12,23	-2,7	0,0001	0,1896	LTBP1
TC0700006844.hg.1	11,15	12,59	-2,7	0,0206	0,7365	ITGB8
TC0100016794.hg.1	11,18	12,6	-2,67	0,0229	0,744	B3GALT2
TC0500012138.hg.1	9,16	10,57	-2,66	0,0002	0,1952	SPOCK1
TC0600009843.hg.1	6,1	7,47	-2,58	0,0014	0,4759	PLEKHG1
TC0900009859.hg.1	6,53	7,89	-2,57	0,0011	0,4402	AQP7
TC1100009954.hg.1	5,61	6,97	-2,57	0,034	0,7831	OR56A1
TC1800007198.hg.1	10,94	12,3	-2,56	0,0003	0,2037	SLC14A1
TC1900008931.hg.1	4,69	6,03	-2,54	0,0052	0,6757	RFPL4A
TC1200010559.hg.1	10,21	11,55	-2,54	0,0477	0,8079	VDR
TC1900006466.hg.1	4,03	5,37	-2,53	0,0023	0,5304	CDC34
TC0800010936.hg.1	9,61	10,95	-2,53	0,0063	0,6955	PMP2
TC0300011550.hg.1	9,13	10,45	-2,5	0,0008	0,4006	PDZRN3
TC0100011333.hg.1	8,22	9,54	-2,49	0,0021	0,5304	CDK18
TC0200010577.hg.1	6,83	8,14	-2,48	0,0132	0,7253	ADAM23
TC0200008749.hg.1	5,85	7,15	-2,46	0,0009	0,4068	C2orf40

TC0500006613.hg.1	5,22	6,51	-2,46	0,0065	0,6972	ADAMTS16
TC0900008982.hg.1	7,44	8,74	-2,45	0,0081	0,7253	AIF1L
TC0100008637.hg.1	4,11	5,39	-2,43	0,007	0,7181	TCTEX1D1
TC0100006486.hg.1	8,77	10,05	-2,43	0,0177	0,7275	AGRN
TC1000009057.hg.1	8,45	9,72	-2,42	0,0005	0,321	NANOS1
TC1700012405.hg.1	7,93	9,21	-2,42	0,0136	0,7253	KRTAP4-8
TC0700009482.hg.1	4,16	5,43	-2,41	9,98E-05	0,1561	TAS2R39
TC0X00009341.hg.1	9,19	10,46	-2,41	0,0082	0,7253	DMD
TC1700011263.hg.1	5,63	6,89	-2,39	0,0075	0,7211	TEX14
TC0700008495.hg.1	6,13	7,39	-2,38	0,0014	0,4759	BUD31
TC1000011026.hg.1	8,72	9,95	-2,36	0,0002	0,1896	USP54
TC1000011904.hg.1	8,73	9,97	-2,36	0,0022	0,5304	ABLIM1
TC1300006599.hg.1	5,54	6,79	-2,36	0,0031	0,5994	C1QTNF9B-AS1
TC1700007511.hg.1	4,78	6,01	-2,35	0,0356	0,788	RHBDL3
TC0700011797.hg.1	11,15	12,38	-2,34	0,0185	0,7275	SAMD9L
TC1100013045.hg.1	4,95	6,17	-2,33	0,0051	0,6757	SIPA1
TC1600008219.hg.1	4,87	6,09	-2,33	0,0371	0,7901	CHTF8
TC0200016424.hg.1	6,96	8,17	-2,32	0,0008	0,4006	LBH
TC1100007111.hg.1	6,73	7,94	-2,32	0,0023	0,5304	BBOX1
TC1400008056.hg.1	7,51	8,71	-2,31	0,0002	0,1952	IFI27
TC0800011064.hg.1	5,88	7,09	-2,31	0,0214	0,7365	CALB1
TC0100008815.hg.1	10,44	11,64	-2,3	0,0252	0,7579	IFI44L
TC1800007508.hg.1	5,09	6,27	-2,28	0,0009	0,4068	TNFRSF11A
TC0600007346.hg.1	5,57	6,75	-2,27	0,0097	0,7253	VN1R10P
TC1700009730.hg.1	9,72	10,89	-2,26	0,0035	0,6181	RCVRN
TC1000010172.hg.1	9,25	10,43	-2,26	0,0038	0,6193	SVIL
TC0400008534.hg.1	8	9,18	-2,26	0,0053	0,6757	NDST3
TC0400010785.hg.1	11,64	12,82	-2,26	0,0098	0,7253	IGFBP7
TC1900007952.hg.1	6,71	7,88	-2,26	0,0165	0,7253	LOC728485; CTD-2162K18.4
TC0100016081.hg.1	4,37	5,54	-2,25	0,0312	0,7831	OR6N2
TC0600012932.hg.1	6,06	7,22	-2,24	0,0395	0,8036	HS3ST5
TC1000012169.hg.1	14,91	16,07	-2,23	0,0023	0,5304	ADAM12
TC0100007017.hg.1	4,95	6,1	-2,23	0,0119	0,7253	C1orf64
TC0500012459.hg.1	9,33	10,49	-2,23	0,0084	0,7253	PDGFRB
TC0100011262.hg.1	12,03	13,18	-2,22	0,0009	0,4068	PRELP

TC1200009157.hg.1	9,17	10,32	-2,22	0,0137	0,7253	P2RX7
TC0300006564.hg.1	5,85	6,99	-2,21	0,0002	0,1952	IL17RE
TC0600012709.hg.1	10,05	11,19	-2,2	0,012	0,7253	POPDC3
TC1200010968.hg.1	6,92	8,05	-2,19	0,0021	0,5304	DDIT3
TC1400007201.hg.1	8,05	9,18	-2,19	0,0133	0,7253	CDKN3
TC1200009829.hg.1	5,12	6,24	-2,18	0,044	0,8056	CLEC4E
TC1300008760.hg.1	5,38	6,49	-2,16	0,0026	0,5632	EPSTI1
TC0200014785.hg.1	10,13	11,24	-2,16	0,0053	0,6757	FIGN
TC1100011886.hg.1	4,32	5,42	-2,15	0,0321	0,7831	RAB38
TC1200012143.hg.1	8,52	9,61	-2,14	0,0027	0,5699	CIT; MIR1178
TC1100012753.hg.1	5,97	7,07	-2,14	0,0125	0,7253	KIRREL3
TC0900007752.hg.1	5,54	6,64	-2,14	0,0176	0,727	NTRK2
TC0500013373.hg.1	8,23	9,33	-2,14	0,036	0,7901	SRA1
TC0600009310.hg.1	9,68	10,77	-2,13	0,0071	0,7196	GJA1
TC1700012039.hg.1	7,89	8,98	-2,13	0,0127	0,7253	LOC100130370; RP11-1055B8.[illegible]
TC0900010445.hg.1	5,31	6,39	-2,12	0,0266	0,76	NMRK1
TC1900008415.hg.1	8,42	9,5	-2,11	0,0161	0,7253	CCDC9
TC1200012149.hg.1	10,15	11,23	-2,1	0,0065	0,6972	GCN1; MIR4498
TC0500011712.hg.1	5,06	6,12	-2,08	0,0036	0,6181	EPB41L4A
TC1900010466.hg.1	12,15	13,21	-2,08	0,0035	0,6181	LGI4
TC2100007420.hg.1	8,14	9,2	-2,08	0,0057	0,6757	COL18A1
TC0700006619.hg.1	8,91	9,97	-2,08	0,012	0,7253	SLC29A4
TC0600008787.hg.1	4,07	5,12	-2,08	0,0349	0,7875	BACH2
TC0100007406.hg.1	8,33	9,38	-2,07	0,0003	0,22	LDLRAP1
TC0300012773.hg.1	13,43	14,48	-2,07	0,0019	0,5304	TM4SF1
TC0900010809.hg.1	6,32	7,38	-2,07	0,0319	0,7831	BICD2
TC0900010388.hg.1	7,9	8,95	-2,06	0,0008	0,4006	TMEM2
TC0700007034.hg.1	14,38	15,42	-2,06	0,0153	0,7253	CREB5
TC0300009856.hg.1	8,03	9,06	-2,05	0,0106	0,7253	IL1RAP
TC0600014055.hg.1	7,25	8,28	-2,05	0,0154	0,7253	NQO2
TC0100018146.hg.1	8,73	9,77	-2,05	0,022	0,7365	ZNF692
TC0100018399.hg.1	7,76	8,79	-2,04	0,0021	0,5304	PLEKHG5
TC0200015242.hg.1	14,88	15,91	-2,04	0,0257	0,7579	STAT1
TC0400012864.hg.1	3,92	4,95	-2,04	0,0337	0,7831	ADAM29
TC0300008106.hg.1	3,69	4,72	-2,04	0,05	0,8086	OR5H2

TC0100006849.hg.1	8,08	9,11	-2,03	0,0005	0,321	ANGPTL7
TC0200016689.hg.1	6,97	7,99	-2,03	0,0008	0,4006	SLC4A5
TC1500006925.hg.1	11,01	12,02	-2,03	0,0011	0,4342	THBS1
TC0100014776.hg.1	10,76	11,78	-2,03	0,0028	0,5772	DDAH1
TC0100015436.hg.1	5,54	6,56	-2,03	0,0095	0,7253	SPAG17
TC0100013123.hg.1	10,15	11,17	-2,03	0,0139	0,7253	UBR4
TC1500010942.hg.1	5,36	6,38	-2,02	0,0015	0,4877	LYSMD4
TC2100008526.hg.1	5,14	6,16	-2,02	0,0016	0,491	SLC37A1
TC0400012990.hg.1	10,74	11,76	-2,02	0,003	0,5958	DDX60L
TC0100018294.hg.1	4,43	5,44	-2,02	0,0068	0,7102	LCE3C
TC1900006508.hg.1	9,68	10,69	-2,02	0,0078	0,7253	ABCA7
TC1100007216.hg.1	10,25	11,27	-2,02	0,0228	0,744	PRRG4
TC0700006690.hg.1	6,16	7,18	-2,02	0,0263	0,7579	NXPH1
TC1100011259.hg.1	7,38	8,39	-2,02	0,0373	0,7908	FOSL1
TC0600011508.hg.1	7,1	8,11	-2,01	0,0061	0,6918	TAP1
TC1900010743.hg.1	9,54	10,55	-2,01	0,0157	0,7253	TGFB1
TC1000006768.hg.1	8,07	9,08	-2,01	0,0421	0,8056	CELF2
TC0600014109.hg.1	4,81	5,81	-2	0,0078	0,7253	PSMB8-AS1
TC0800012389.hg.1	4,69	5,68	-1,99	0,0072	0,7211	MSR1
TC2000006625.hg.1	5,71	6,71	-1,99	0,0087	0,7253	C20orf196
TC0700010435.hg.1	10,07	11,06	-1,99	0,0134	0,7253	CDCA7L
TC0400011203.hg.1	5,59	6,58	-1,99	0,0104	0,7253	HPSE
TC1600007965.hg.1	5,84	6,83	-1,99	0,0107	0,7253	MT1F
TC0500008086.hg.1	8,56	9,55	-1,99	0,0165	0,7253	NR2F1
TC0700011586.hg.1	7,57	8,56	-1,99	0,0242	0,7517	GSAP
TC1500010737.hg.1	7,58	8,57	-1,98	0,0014	0,4877	SQRDL
TC0200016649.hg.1	4,28	5,27	-1,98	0,0137	0,7253	CDC42EP3
TC0200016623.hg.1	7,69	8,67	-1,98	0,0167	0,7253	KIDINS220
TC1500010350.hg.1	5,39	6,38	-1,98	0,0405	0,8036	NTRK3
TC1600006561.hg.1	10,59	11,57	-1,97	0,0073	0,7211	MAPK8IP3
TC1700010653.hg.1	7,69	8,66	-1,97	0,0254	0,7579	KRTAP2-3
TC1900009100.hg.1	6,46	7,44	-1,97	0,0257	0,7579	POLRMT
TC0300013965.hg.1	4,96	5,93	-1,97	0,0464	0,8056	PRSS46
TC0200016402.hg.1	7,75	8,72	-1,96	0,0052	0,6757	RSAD2
TC1900008879.hg.1	4,52	5,49	-1,96	0,0153	0,7253	KIR2DS5

TC2200008818.hg.1	7,04	8,01	-1,96	0,0303	0,7826	CHADL
TC0X00009203.hg.1	4,46	5,44	-1,96	0,047	0,8075	MAP3K15
TC1100011514.hg.1	10,87	11,83	-1,96	0,0483	0,8079	DHCR7
TC1500008236.hg.1	8,72	9,69	-1,95	0,004	0,6441	ACAN
TC1500007546.hg.1	8,74	9,7	-1,95	0,0108	0,7253	SNX22
TC1600006658.hg.1	6,68	7,64	-1,95	0,0191	0,7283	IL32
TC0600012116.hg.1	4,42	5,38	-1,95	0,0202	0,7365	COL21A1
TC0500010453.hg.1	6,19	7,15	-1,95	0,0278	0,768	ADAMTS12
TC0200008680.hg.1	4,21	5,16	-1,94	0,0009	0,4068	SLC9A2
TC0600014135.hg.1	3,57	4,52	-1,94	0,0012	0,4402	ADGRF2
TC0700010155.hg.1	7,34	8,3	-1,94	0,0053	0,6757	TNRC18
TC0600007303.hg.1	10,02	10,97	-1,94	0,0183	0,7275	BTN3A1
TC0100013445.hg.1	13,22	14,18	-1,94	0,0184	0,7275	IFI6
TC1200012708.hg.1	9,79	10,75	-1,94	0,0288	0,7733	OAS1
TC1200012593.hg.1	6,12	7,07	-1,93	0,0032	0,5994	CLEC4A
TC1400008423.hg.1	6,01	6,95	-1,93	0,0036	0,6181	TDRD9
TC0X00008674.hg.1	4,95	5,89	-1,93	0,0075	0,7211	AFF2
TC1000012512.hg.1	7,11	8,05	-1,93	0,0143	0,7253	PAOX
TC0300011019.hg.1	8,27	9,21	-1,93	0,0167	0,7253	PLXNB1
TC1300008858.hg.1	5,19	6,14	-1,93	0,0371	0,7901	HTR2A
TC1700007634.hg.1	7,06	8,01	-1,93	0,036	0,7901	ZNHIT3
TC1000007272.hg.1	9,94	10,88	-1,92	0,0061	0,6918	CREM
TC0700013355.hg.1	3,93	4,87	-1,92	0,0061	0,6918	NME8
TC0900012140.hg.1	7,67	8,61	-1,92	0,0267	0,7621	PGM5
TC1700011007.hg.1	9,3	10,23	-1,92	0,0337	0,7831	TTLL6
TC2000006861.hg.1	14,61	15,55	-1,92	0,0478	0,8079	RIN2
TC2200006985.hg.1	6	6,93	-1,91	0,0026	0,5632	PITPNB
TC1600010396.hg.1	3,98	4,92	-1,91	0,0055	0,6757	CES5A
TC1200008920.hg.1	10,01	10,94	-1,91	0,0123	0,7253	OAS3
TC1100012651.hg.1	4,64	5,57	-1,91	0,0119	0,7253	OR10G7
TC0500009424.hg.1	5,5	6,44	-1,91	0,0194	0,7283	FGF18
TC0200016744.hg.1	4,18	5,11	-1,91	0,0247	0,7537	ITGB6
TC1600006637.hg.1	8,28	9,21	-1,91	0,0255	0,7579	ZG16B
TC1900008103.hg.1	11,72	12,65	-1,9	0,0027	0,5699	PLD3
TC2200007909.hg.1	12,73	13,66	-1,9	0,0075	0,7211	MICAL3

TC0100007791.hg.1	6,95	7,87	-1,9	0,0259	0,7579	ADPRHL2
TC1100012712.hg.1	6,44	7,37	-1,9	0,0287	0,7733	ACRV1
TC0400012977.hg.1	12,63	13,55	-1,89	0,002	0,5304	SH3D19
TC1800008304.hg.1	5,33	6,25	-1,89	0,0124	0,7253	OSBPL1A
TC0300008561.hg.1	12,42	13,34	-1,89	0,0099	0,7253	PARP14
TC1600011545.hg.1	8,29	9,21	-1,89	0,015	0,7253	PLLP
TC0600013186.hg.1	13,96	14,88	-1,88	0,0088	0,7253	CTGF
TC0600008515.hg.1	6,48	7,39	-1,88	0,0127	0,7253	KCNQ5
TC1900007026.hg.1	4,53	5,44	-1,88	0,0269	0,7645	SWSAP1
TC0600014081.hg.1	6,46	7,36	-1,88	0,0283	0,7719	HFE
TC0100012359.hg.1	7,18	8,09	-1,88	0,0321	0,7831	OR2T3
TC0100018309.hg.1	10,08	10,99	-1,88	0,0431	0,8056	FCGR2C
TC0100009866.hg.1	5,29	6,19	-1,87	0,0055	0,6757	FCGR1A
TC0800007051.hg.1	4,45	5,35	-1,87	0,0049	0,6757	NEFM
TC0700009647.hg.1	7,36	8,26	-1,87	0,0095	0,7253	ZNF862
TC2200009236.hg.1	8,65	9,55	-1,86	0,002	0,5304	GGT1
TC0400007848.hg.1	5,51	6,41	-1,86	0,0056	0,6757	MTHFD2L
TC0100009221.hg.1	10,93	11,83	-1,86	0,0065	0,6972	VCAM1
TC0300009799.hg.1	7,97	8,87	-1,86	0,0088	0,7253	RTP4
TC0600009800.hg.1	11,6	12,49	-1,86	0,019	0,7283	GINM1
TSUnmapped000006	7,79	8,67	-1,85	0,002	0,5304	MLXIP
TC2000009060.hg.1	5,65	6,54	-1,85	0,0043	0,6489	KIAA1755
TC1900007150.hg.1	11,12	12	-1,85	0,0169	0,7253	CC2D1A
TC0200016668.hg.1	9,17	10,05	-1,85	0,0189	0,7283	GPR75
TC1900007859.hg.1	3,35	4,24	-1,85	0,0253	0,7579	FFAR2
TC1400006494.hg.1	4,41	5,29	-1,85	0,0322	0,7831	OR4K15
TC0200014772.hg.1	10,48	11,36	-1,85	0,0369	0,7901	IFIH1
TC1000011607.hg.1	3,51	4,39	-1,84	0,0038	0,6193	PKD2L1
TC0800009486.hg.1	5,54	6,42	-1,84	0,0142	0,7253	DEFB107A; DEFB107B
TC1200010221.hg.1	4,77	5,65	-1,84	0,0093	0,7253	MANSC4
TC1600011466.hg.1	5,93	6,81	-1,84	0,0134	0,7253	PKMYT1
TC0600014260.hg.1	6,5	7,38	-1,84	0,0223	0,7371	ATP6V1G2
TC2200006637.hg.1	9,91	10,79	-1,84	0,0426	0,8056	DGCR8
TC2200007505.hg.1	11,02	11,9	-1,83	0,0009	0,4068	03-set
TC0200008675.hg.1	6,04	6,91	-1,83	0,0053	0,6757	IL18RAP

TC0300008082.hg.1	4,46	5,34	-1,83	0,0127	0,7253	EPHA6
TSUnmapped000002	9,43	10,3	-1,83	0,0093	0,7253	PSMC4
TC0300013146.hg.1	5,75	6,63	-1,83	0,039	0,8002	TNFSF10
TC0100012334.hg.1	4,32	5,18	-1,82	0,0016	0,491	OR2L8
TC0400006773.hg.1	6,23	7,09	-1,82	0,0148	0,7253	LOC650293
TC0100010255.hg.1	5,04	5,9	-1,82	0,0079	0,7253	OR10J4
TC0700013581.hg.1	5,27	6,13	-1,82	0,0155	0,7253	STEAP4
TC1900007748.hg.1	9,38	10,25	-1,82	0,0188	0,7283	PDCD5
TC1700010645.hg.1	4,81	5,68	-1,82	0,0266	0,76	KRTAP3-2
TC0100014013.hg.1	5,6	6,46	-1,82	0,0397	0,8036	CCDC163P
TC0100009846.hg.1	6,15	7	-1,81	0,0029	0,5772	PDE4DIP
TC0800010282.hg.1	5,03	5,89	-1,81	0,005	0,6757	DKK4
TC0300012191.hg.1	11,36	12,22	-1,81	0,007	0,7181	PARP9
TC0100015866.hg.1	9,77	10,63	-1,81	0,0185	0,7275	S100A4
TC1100013030.hg.1	7,43	8,29	-1,81	0,0306	0,7826	FLRT1
TC1100008392.hg.1	11,4	12,24	-1,8	0,0036	0,6181	PLEKHB1
TC0600010802.hg.1	9,1	9,95	-1,8	0,0174	0,7253	ELOVL2
TC0500006744.hg.1	5,02	5,87	-1,8	0,0156	0,7253	ROPN1L
TC0600012763.hg.1	4,71	5,56	-1,8	0,0097	0,7253	SCML4
TC2200009270.hg.1	7,44	8,29	-1,8	0,0216	0,7365	APOBEC3B
TC0800006739.hg.1	6,79	7,64	-1,8	0,0274	0,7679	SLC35G5
TC0100013634.hg.1	7,71	8,56	-1,79	0,0065	0,6972	TMEM54
TC0900006559.hg.1	6,06	6,9	-1,79	0,0121	0,7253	CD274
TC1900007862.hg.1	4,67	5,51	-1,79	0,0139	0,7253	GAPDHS
TC0100015194.hg.1	11,97	12,81	-1,79	0,0155	0,7253	SORT1
TC1100009617.hg.1	3,52	4,37	-1,79	0,0235	0,7517	GLB1L3
TC0200016563.hg.1	7,69	8,52	-1,79	0,0279	0,768	KLHL23
TC0600007613.hg.1	10,61	11,44	-1,78	0,0102	0,7253	HSPA1A; HSPA1B
TC0500012942.hg.1	7,4	8,23	-1,78	0,0157	0,7253	RNF44
TC0200008291.hg.1	8,26	9,09	-1,78	0,041	0,8036	KDM3A
TC0500011347.hg.1	12,39	13,21	-1,77	0,0014	0,4759	HAPLN1
TC0200011888.hg.1	9,69	10,51	-1,77	0,0027	0,5699	MATN3
TC0800008679.hg.1	8,23	9,05	-1,77	0,0046	0,6723	COL14A1
TC0600012560.hg.1	3,63	4,46	-1,77	0,0097	0,7253	BACH2
TC1000008582.hg.1	4,39	5,21	-1,77	0,0109	0,7253	C10orf62

TC0900008963.hg.1	7,69	8,51	-1,77	0,0143	0,7253	FUBP3
TC1400010586.hg.1	7,63	8,45	-1,77	0,0168	0,7253	LTB4R; LTB4R2
TC1100012130.hg.1	5,5	6,32	-1,77	0,0197	0,7338	MMP10
TC1900007947.hg.1	5,76	6,58	-1,77	0,0219	0,7365	ZNF382
TC1700007645.hg.1	6,15	6,97	-1,77	0,0227	0,744	CTB-75G16.3; MRM1
TC0300009310.hg.1	7,16	7,99	-1,77	0,0263	0,7579	MLF1
TC0100010043.hg.1	10,08	10,89	-1,76	0,0012	0,4402	S100A1
TC2200007069.hg.1	8,18	9	-1,76	0,0055	0,6757	OSBP2
TC1900008416.hg.1	4,71	5,53	-1,76	0,007	0,7181	INAFM1
TC0700008760.hg.1	10,21	11,02	-1,76	0,0074	0,7211	SLC26A4
TC1900011984.hg.1	8,28	9,09	-1,76	0,0118	0,7253	DMPK
TSUnmapped000001	4,52	5,34	-1,76	0,0091	0,7253	SAG
TC0300013831.hg.1	7,36	8,18	-1,76	0,017	0,7253	SEMA3B; MIR6872
TC1100013084.hg.1	11,65	12,47	-1,76	0,0251	0,7579	AMOTL1
TC0X00007183.hg.1	4,08	4,89	-1,76	0,0274	0,7679	SSX4B; SSX4
TC0400011313.hg.1	10,75	11,57	-1,76	0,0303	0,7826	SNCA
TC1100010838.hg.1	4,74	5,54	-1,75	0,0023	0,5304	OR5F1
TC0800008300.hg.1	9,25	10,06	-1,75	0,0042	0,6489	CPQ
TC0100009560.hg.1	7,92	8,72	-1,75	0,0159	0,7253	CD101
TC2200007662.hg.1	8,64	9,45	-1,75	0,0172	0,7253	GTSE1
TC1700008261.hg.1	5,34	6,15	-1,75	0,0185	0,7275	EPN3
TC0400008105.hg.1	10,63	11,43	-1,75	0,0205	0,7365	HERC6
TC1900009382.hg.1	12,74	13,55	-1,75	0,0407	0,8036	PTPRS
TC1700009566.hg.1	6,85	7,65	-1,74	0,0043	0,6489	NLRP1
TC0100011064.hg.1	5,65	6,46	-1,74	0,0083	0,7253	CFH
TC0600012713.hg.1	12,07	12,86	-1,74	0,0108	0,7253	PREP
TC0700007345.hg.1	12,61	13,41	-1,74	0,0158	0,7253	STK17A
TC1900010538.hg.1	6,43	7,23	-1,74	0,0091	0,7253	ZNF790
TC0400006984.hg.1	4,6	5,4	-1,74	0,022	0,7365	CLRN2
TC0900012055.hg.1	7,29	8,1	-1,74	0,0213	0,7365	EXD3
TC1900009682.hg.1	8,03	8,83	-1,74	0,0221	0,7371	ELOF1
TC2100007593.hg.1	4,82	5,62	-1,74	0,0258	0,7579	LIPI
TC0100016988.hg.1	12,42	13,22	-1,74	0,0457	0,8056	FMOD
TC0500012039.hg.1	7,84	8,64	-1,74	0,0465	0,8056	ZCCHC10
TC0600010057.hg.1	7,43	8,22	-1,73	0,0034	0,6181	ACAT2

TC1700012158.hg.1	7,71	8,5	-1,73	0,0129	0,7253	B3GNTL1
TC0300007973.hg.1	4,88	5,67	-1,73	0,0089	0,7253	CADM2
TC1600011399.hg.1	10,68	11,47	-1,73	0,0154	0,7253	MT1X
TC1100009212.hg.1	11,02	11,82	-1,73	0,0117	0,7253	PHLDB1; MIR6716
TC2200009314.hg.1	7,79	8,58	-1,73	0,0214	0,7365	RTN4R
TC1700007929.hg.1	9,99	10,78	-1,73	0,0276	0,7679	RUNDC1
TC2200007331.hg.1	10,13	10,92	-1,73	0,0282	0,7719	PICK1
TC0300007596.hg.1	7,27	8,07	-1,73	0,0297	0,7826	FLNB
TC1600010801.hg.1	3,64	4,43	-1,73	0,0301	0,7826	PKD1L3
TC1700012057.hg.1	6,35	7,14	-1,73	0,0353	0,788	OXLD1
TSUnmapped000001	7,87	8,67	-1,73	0,0362	0,7901	DUSP16
TC1100009951.hg.1	4,54	5,33	-1,73	0,0495	0,8086	OR52L1
TC1900008090.hg.1	7,01	7,79	-1,72	0,0042	0,6489	MAP3K10
TC1900012050.hg.1	10,37	11,15	-1,72	0,0062	0,6955	PPP6R1
TC2200009366.hg.1	9,33	10,12	-1,72	0,0085	0,7253	CHKB-CPT1B
TC0700012708.hg.1	7,79	8,57	-1,72	0,0084	0,7253	SLC13A4
TC0800009752.hg.1	12,14	12,92	-1,72	0,0212	0,7365	ASAH1
TC1500008170.hg.1	6,74	7,52	-1,72	0,0244	0,7517	ALPK3
TC0900008467.hg.1	7,24	8,02	-1,72	0,0244	0,7517	KIAA1958
TC1700010019.hg.1	6,65	7,44	-1,72	0,0262	0,7579	MFAP4
TC1600011372.hg.1	9,54	10,32	-1,72	0,0465	0,8056	SULT1A4; SULT1A3
TC0200011467.hg.1	7,77	8,55	-1,72	0,0481	0,8079	TMEM18
TC1900006576.hg.1	8,68	9,47	-1,72	0,0488	0,8086	DOT1L
TC1900006892.hg.1	4,17	4,94	-1,71	0,0128	0,7253	ANGPTL4
TC0400012182.hg.1	3,55	4,32	-1,71	0,0089	0,7253	FGG
TC0300007161.hg.1	6,3	7,07	-1,71	0,0158	0,7253	VIPR1
TC1900007975.hg.1	3,77	4,54	-1,71	0,014	0,7253	ZNF570
TC1100012949.hg.1	12,58	13,36	-1,71	0,0183	0,7275	IFITM1
TC1200009148.hg.1	3,74	4,51	-1,71	0,0225	0,7412	HNF1A
TC0800011132.hg.1	7,68	8,46	-1,71	0,0485	0,8079	GEM
TC1900009134.hg.1	11,98	12,74	-1,7	0,0153	0,7253	SBNO2
TC1900011061.hg.1	9,39	10,16	-1,7	0,0273	0,7679	CARD8
TC1100009967.hg.1	5,19	5,95	-1,7	0,0322	0,7831	PRKCDBP
TC0700008928.hg.1	7,21	7,97	-1,69	0,0091	0,7253	CPED1
TC0100008912.hg.1	8,28	9,04	-1,69	0,0145	0,7253	CYR61

TC0400008977.hg.1	6,3	7,06	-1,69	0,0125	0,7253	MAB21L2
TC0200009662.hg.1	10,31	11,07	-1,69	0,0151	0,7253	TNFAIP6
TC0200008984.hg.1	10,65	11,41	-1,69	0,0257	0,7579	RABL2A
TC0300011065.hg.1	7,11	7,86	-1,69	0,0375	0,7908	AMT; NICN1
TC1100010948.hg.1	4,87	5,63	-1,69	0,0409	0,8036	GLYAT
TC0700010035.hg.1	7,61	8,37	-1,69	0,0413	0,8036	MAD1L1
TC0800010426.hg.1	4,7	5,45	-1,69	0,0442	0,8056	FAM150A
TC0200006936.hg.1	7,39	8,14	-1,68	0,0042	0,6489	KLHL29
TC1900008358.hg.1	8,33	9,07	-1,68	0,0159	0,7253	CCDC61
TC2100007452.hg.1	6,75	7,49	-1,68	0,0123	0,7253	COL6A2
TC0400006833.hg.1	5,42	6,17	-1,68	0,0091	0,7253	DEFB131
TC1500010931.hg.1	6,31	7,06	-1,68	0,0085	0,7253	FAM174B
TC0200010264.hg.1	9,54	10,3	-1,68	0,0105	0,7253	MFSD6
TC0300009258.hg.1	3,61	4,36	-1,68	0,0144	0,7253	MME
TC0200014672.hg.1	10,29	11,04	-1,68	0,0112	0,7253	NR4A2
TC0600007387.hg.1	6,32	7,07	-1,68	0,0097	0,7253	OR2B6
TC0900010933.hg.1	5,31	6,06	-1,68	0,0208	0,7365	CTSV
TC1100013081.hg.1	5,4	6,15	-1,68	0,0261	0,7579	FUT4
TC0500010635.hg.1	10,65	11,39	-1,68	0,0275	0,7679	HMGCS1
TC1900009592.hg.1	11,26	12,01	-1,68	0,032	0,7831	ZNF562
TC1300009673.hg.1	7,25	7,99	-1,68	0,0362	0,7901	TEX30
TC1600011373.hg.1	8,76	9,5	-1,68	0,046	0,8056	SLX1A-SULT1A3
TC0100018310.hg.1	9,3	10,05	-1,67	0,0091	0,7253	FCGR2B
TC1200008667.hg.1	8,84	9,58	-1,67	0,0108	0,7253	HSP90B1
TC0100010140.hg.1	10,59	11,33	-1,67	0,0161	0,7253	SYT11
TC0200007421.hg.1	7,09	7,83	-1,67	0,0186	0,7275	CAMKMT
TC0100014276.hg.1	10,73	11,47	-1,67	0,0213	0,7365	DHCR24
TC1400008645.hg.1	5,78	6,52	-1,67	0,0248	0,7555	OR4E1
TC0100018531.hg.1	7,62	8,36	-1,67	0,0342	0,7842	PIGC
TC0200016302.hg.1	5,69	6,43	-1,67	0,0349	0,7875	PRR21
TC0700012230.hg.1	7,3	8,05	-1,67	0,0376	0,7908	NRCAM
TC2200007952.hg.1	8,65	9,38	-1,66	0,0039	0,629	CLTCL1
TC1200012835.hg.1	9,94	10,68	-1,66	0,0052	0,6757	NT5DC3
TC0600012314.hg.1	14,88	15,61	-1,66	0,006	0,6896	COL12A1
TC0100010024.hg.1	5,39	6,12	-1,66	0,0059	0,6896	LELP1

TC0400013004.hg.1	5,03	5,76	-1,66	0,0081	0,7253	MTNR1A
TC1900009947.hg.1	8,66	9,39	-1,66	0,0168	0,7253	MYO9B
TC0900011977.hg.1	7,6	8,33	-1,66	0,0129	0,7253	SNAPC4
TC1600010789.hg.1	4,71	5,44	-1,66	0,0094	0,7253	TAT
TC0X00008147.hg.1	3,35	4,08	-1,66	0,0154	0,7253	ZCCHC16
TC0100014958.hg.1	8,5	9,23	-1,66	0,0215	0,7365	BCAR3
TC1100009284.hg.1	6,5	7,23	-1,66	0,0217	0,7365	POU2F3
TC0X00008836.hg.1	8,51	9,24	-1,66	0,0233	0,7517	PLXNA3
TC0100018185.hg.1	11	11,73	-1,66	0,0256	0,7579	MINOS1
TC0600011777.hg.1	5,71	6,44	-1,66	0,0435	0,8056	TREM1
TC1700009476.hg.1	3,68	4,42	-1,66	0,0491	0,8086	OR3A1
TC1900006989.hg.1	8,46	9,19	-1,65	0,0131	0,7253	ATG4D
TC0200009700.hg.1	8,67	9,39	-1,65	0,009	0,7253	GALNT13
TC0400009088.hg.1	9,57	10,29	-1,65	0,0142	0,7253	GUCY1B3
TC2200009198.hg.1	7,34	8,07	-1,65	0,0146	0,7253	P2RX6
TC0100018321.hg.1	6,24	6,96	-1,65	0,0215	0,7365	TEX35
TC0900008865.hg.1	12,45	13,18	-1,65	0,0229	0,7445	CERCAM
TC1100008330.hg.1	9,45	10,17	-1,65	0,0407	0,8036	IL18BP
TC1100009279.hg.1	9,41	10,12	-1,64	0,0066	0,6999	OAF
TC0500010523.hg.1	10,04	10,75	-1,64	0,0144	0,7253	GDNF
TC0100015871.hg.1	9,53	10,24	-1,64	0,0095	0,7253	S100A16
TC1100011833.hg.1	8,82	9,53	-1,64	0,0159	0,7253	SYTL2
TC0X00011395.hg.1	5,17	5,89	-1,64	0,0124	0,7253	USP26
TC1900009670.hg.1	7,84	8,55	-1,64	0,0193	0,7283	DOCK6
TC1200009489.hg.1	6,87	7,59	-1,64	0,0228	0,744	FBRSL1
TC1900006969.hg.1	10,71	11,42	-1,64	0,024	0,7517	PPAN-P2RY11;
TC0900008499.hg.1	6,79	7,51	-1,64	0,0245	0,7517	ZNF618
TC0200013376.hg.1	9,82	10,54	-1,64	0,0265	0,7593	EIF2AK3
TC0300012818.hg.1	4,61	5,32	-1,64	0,0408	0,8036	IGSF10
TC0600014253.hg.1	5,47	6,18	-1,64	0,0409	0,8036	OR5V1
TC0100006865.hg.1	7,44	8,15	-1,64	0,0433	0,8056	AGTRAP
TC0700007374.hg.1	12,49	13,21	-1,64	0,0444	0,8056	YKT6
TC0200016408.hg.1	7,43	8,14	-1,64	0,0469	0,8062	KCNS3
TC0800007057.hg.1	11,28	11,98	-1,63	0,0101	0,7253	DOCK5
TC0X00009101.hg.1	13,78	14,49	-1,63	0,0145	0,7253	GPM6B

TC1400009175.hg.1	7,33	8,04	-1,63	0,0102	0,7253	NID2
TC1600010978.hg.1	5,71	6,41	-1,63	0,0165	0,7253	PKD1L2
TC1100009453.hg.1	11,39	12,09	-1,63	0,0077	0,7253	ST3GAL4
TC0200014597.hg.1	12,05	12,76	-1,63	0,0278	0,768	RND3
TC1700012184.hg.1	6,96	7,67	-1,63	0,0291	0,7751	CHRNB1
TC1600007992.hg.1	10,95	11,66	-1,63	0,0326	0,7831	ARL2BP
TSUnmapped000000	5,49	6,19	-1,63	0,0313	0,7831	ATG16L1
TC1100007519.hg.1	5,41	6,11	-1,63	0,0334	0,7831	OR4C3
TC0100008816.hg.1	10,42	11,13	-1,63	0,036	0,7901	IFI44
TC2000008111.hg.1	6,16	6,86	-1,63	0,038	0,7938	MYT1
TC1700012406.hg.1	6,14	6,84	-1,63	0,0455	0,8056	KRTAP4-11
TC0400011234.hg.1	6,44	7,15	-1,63	0,0442	0,8056	MAPK10
TC0700007111.hg.1	5,66	6,36	-1,62	0,0054	0,6757	CCDC129
TC0900009825.hg.1	9,72	10,42	-1,62	0,0142	0,7253	DDX58
TC1000011933.hg.1	8,24	8,93	-1,62	0,0122	0,7253	HSPA12A
TSUnmapped000000	6,28	6,97	-1,62	0,0113	0,7253	HYOU1
TC0400010935.hg.1	6,1	6,8	-1,62	0,0172	0,7253	UGT2B4
TC0X00007790.hg.1	4,92	5,61	-1,62	0,019	0,7283	DACH2
TC1400007595.hg.1	8,44	9,14	-1,62	0,0192	0,7283	SIPA1L1
TC2100008527.hg.1	6,07	6,77	-1,62	0,0243	0,7517	SLC37A1
TC0200016662.hg.1	13,42	14,12	-1,62	0,0289	0,774	CALM2
TC0300008789.hg.1	4,71	5,41	-1,62	0,0311	0,7831	H1FOO
TC0300009974.hg.1	5,75	6,44	-1,62	0,0346	0,7872	MUC20; SDHAP2; MIR570;
TC0200010713.hg.1	11,25	11,95	-1,62	0,0351	0,7875	ATIC
TC0400006804.hg.1	6,53	7,22	-1,62	0,0378	0,7908	USP17L22; USP17L20
TC0400008680.hg.1	8,06	8,76	-1,62	0,0429	0,8056	JADE1
TC0200010745.hg.1	11,43	12,12	-1,61	0,0038	0,6193	IGFBP2
TC0700012043.hg.1	7,7	8,39	-1,61	0,0044	0,6573	CLDN15
TC0900010355.hg.1	4,39	5,08	-1,61	0,0098	0,7253	C9orf135-AS1
TC0600011880.hg.1	9,96	10,65	-1,61	0,0152	0,7253	GTPBP2
TC0200009902.hg.1	6,19	6,88	-1,61	0,0121	0,7253	NOSTRIN
TC1900009568.hg.1	6,28	6,97	-1,61	0,0089	0,7253	OR7D4
TC0500012236.hg.1	9,35	10,03	-1,61	0,0125	0,7253	PFDN1
TC0200006891.hg.1	10,29	10,98	-1,61	0,0133	0,7253	RHOB
TC0X00007919.hg.1	12,07	12,76	-1,61	0,0158	0,7253	SRPX2

TC1700010230.hg.1	7,94	8,63	-1,61	0,0127	0,7253	TIAF1; MYO18A
TC1900006819.hg.1	10,7	11,38	-1,61	0,0096	0,7253	TRIP10
TC0300007063.hg.1	4,49	5,18	-1,61	0,014	0,7253	VILL
TC0200013112.hg.1	13,2	13,89	-1,61	0,0216	0,7365	AUP1
TC2200009281.hg.1	8,38	9,07	-1,61	0,0253	0,7579	PNPLA3
TC1300007707.hg.1	4,43	5,12	-1,61	0,0327	0,7831	GPC5
TC0100010268.hg.1	6,94	7,62	-1,61	0,0374	0,7908	FCRL6
TC0500009147.hg.1	4,64	5,33	-1,61	0,0428	0,8056	GRIA1
TC0300012924.hg.1	7,18	7,86	-1,61	0,0485	0,8079	VEPH1
TC0800012363.hg.1	9,06	9,74	-1,6	0,0042	0,6489	KHDRBS3
TC2200007508.hg.1	8,77	9,46	-1,6	0,0131	0,7253	FAM109B
TC1700010560.hg.1	10,2	10,88	-1,6	0,0114	0,7253	PLXDC1
TC1900011944.hg.1	8,03	8,71	-1,6	0,0207	0,7365	FBXO17
TC1100010058.hg.1	7,99	8,67	-1,6	0,0242	0,7517	DENND5A
TC0200012219.hg.1	10,87	11,55	-1,6	0,0242	0,7517	FAM98A
TC0500008466.hg.1	3,71	4,39	-1,6	0,0254	0,7579	FTMT
TC0400012126.hg.1	7,96	8,64	-1,6	0,0275	0,7679	GATB
TC0400008380.hg.1	6,5	7,18	-1,6	0,0376	0,7908	CCDC109B
TC1300009993.hg.1	4,29	4,97	-1,6	0,0412	0,8036	BIVM
TC0400011882.hg.1	7,77	8,45	-1,6	0,0426	0,8056	PABPC4L
TC1900008988.hg.1	7,97	8,65	-1,6	0,0454	0,8056	ZNF805
TC0400011531.hg.1	5,41	6,08	-1,6	0,049	0,8086	GIMD1
TC0200007237.hg.1	10,83	11,5	-1,59	0,0044	0,6534	CRIM1
TC1100013065.hg.1	4,49	5,15	-1,59	0,017	0,7253	KRTAP5-9
TC1100008730.hg.1	6,04	6,71	-1,59	0,0172	0,7253	TRIM49C
TC0700010501.hg.1	6,76	7,43	-1,59	0,0182	0,7275	DFNA5
TC0400012378.hg.1	8,54	9,21	-1,59	0,0206	0,7365	SH3RF1
TC0800010379.hg.1	5,51	6,17	-1,59	0,0228	0,744	EFCAB1
TC0200016552.hg.1	4,51	5,18	-1,59	0,0239	0,7517	UPP2
TC0200012073.hg.1	9,68	10,35	-1,59	0,0245	0,7518	GTF3C2
TC0700010857.hg.1	13,59	14,26	-1,59	0,0255	0,7579	GLI3
TC0200016366.hg.1	8,12	8,79	-1,59	0,0259	0,7579	THAP4
TC1900009325.hg.1	11,72	12,39	-1,59	0,0307	0,7826	MAP2K2
TC0700013543.hg.1	11,79	12,45	-1,59	0,0338	0,7831	C7orf25
TC1200007823.hg.1	14,01	14,69	-1,59	0,032	0,7831	RPS26

TC0200008063.hg.1	9,69	10,35	-1,59	0,0375	0,7908	DGUOK
TC1000011588.hg.1	7,16	7,83	-1,59	0,0378	0,7908	SLC25A28
TC0100013368.hg.1	8,06	8,73	-1,59	0,0385	0,7965	PAQR7
TC0300012233.hg.1	11,73	12,39	-1,59	0,04	0,8036	ITGB5
TC0400012796.hg.1	12,43	13,1	-1,59	0,0463	0,8056	PDGFRA
TC1200012153.hg.1	11,32	11,98	-1,59	0,0451	0,8056	PXN
TC1100007619.hg.1	5,96	6,63	-1,59	0,0468	0,8061	TRIM51
TC0300008994.hg.1	9,54	10,22	-1,59	0,0477	0,8079	PXYLP1
TC0100017167.hg.1	9,02	9,68	-1,58	0,0112	0,7253	LAMB3; MIR4260
TC0300013914.hg.1	7,19	7,85	-1,58	0,0135	0,7253	TPRG1
TC0200007999.hg.1	9,33	9,99	-1,58	0,0246	0,7526	ZNF638
TC2100006873.hg.1	5,18	5,84	-1,58	0,0286	0,7719	KRTAP13-1
TC0200012776.hg.1	7,18	7,84	-1,58	0,0307	0,7826	TMEM17
TC0700010789.hg.1	4,51	5,17	-1,58	0,0323	0,7831	AMPH
TC1700010447.hg.1	6,35	7,01	-1,58	0,0335	0,7831	CCL5
TC1000008489.hg.1	6,52	7,18	-1,58	0,0331	0,7831	LGI1
TC0700013525.hg.1	4,95	5,62	-1,58	0,037	0,7901	FAM126A
TC1900008015.hg.1	7,17	7,82	-1,58	0,0456	0,8056	SPRED3
TC0700010189.hg.1	13,36	14,01	-1,57	0,0031	0,5994	CYTH3
TC0X00008844.hg.1	5,37	6,02	-1,57	0,0041	0,6441	IKBKG
TC0100006773.hg.1	7,98	8,63	-1,57	0,0064	0,6972	SPSB1
TC0800011691.hg.1	4,75	5,4	-1,57	0,017	0,7253	ANXA13
TC0100012471.hg.1	6,68	7,34	-1,57	0,0157	0,7253	AURKAIP1
TC1600011422.hg.1	10,44	11,1	-1,57	0,0145	0,7253	CLEC18A
TC0200009189.hg.1	9,64	10,29	-1,57	0,0147	0,7253	GYPC
TC0800006697.hg.1	5,28	5,93	-1,57	0,0164	0,7253	MSRA
TC0200013373.hg.1	3,78	4,43	-1,57	0,02	0,7365	FOXI3
TC0100007675.hg.1	5,09	5,74	-1,57	0,028	0,7695	FAM167B
TC1300007561.hg.1	4,55	5,2	-1,57	0,0304	0,7826	SCEL
TC2200008103.hg.1	8,35	8,99	-1,57	0,0365	0,7901	YPEL1
TC0600009364.hg.1	9,17	9,81	-1,57	0,0409	0,8036	NCOA7
TC0100017708.hg.1	9,27	9,91	-1,57	0,0446	0,8056	C1orf198
TC0500013280.hg.1	5,95	6,59	-1,56	0,0057	0,6757	ZDHHC11B
TC0900008532.hg.1	5,37	6,02	-1,56	0,007	0,7181	01-dic
TC1000007625.hg.1	6,54	7,18	-1,56	0,0159	0,7253	PRKG1

TC0200015476.hg.1	10,38	11,03	-1,56	0,01	0,7253	RAPH1
TC0800012212.hg.1	9,07	9,71	-1,56	0,0133	0,7253	SLC39A4
TC1100007785.hg.1	12,53	13,17	-1,56	0,0192	0,7283	TMEM109
TSUnmapped000005	10,21	10,85	-1,56	0,0214	0,7365	HYOU1
TC2100006968.hg.1	9,24	9,88	-1,56	0,0258	0,7579	IFNGR2
TC0X00011354.hg.1	5,91	6,55	-1,56	0,0265	0,7593	FIGF
TC1800008781.hg.1	5,98	6,62	-1,56	0,0334	0,7831	ATP8B1
TC0600007701.hg.1	8,66	9,31	-1,56	0,0323	0,7831	PHF1
TC0100012332.hg.1	5,07	5,71	-1,56	0,0388	0,7993	OR2L13
TC2200008356.hg.1	10,91	11,55	-1,56	0,039	0,8	TTC28
TC0200016466.hg.1	6,26	6,9	-1,56	0,041	0,8036	APLF
TC0500006472.hg.1	9,21	9,85	-1,56	0,0445	0,8056	TRIP13
TC0600013295.hg.1	3,76	4,39	-1,55	0,0138	0,7253	IL20RA
TC0200014610.hg.1	9,33	9,97	-1,55	0,0124	0,7253	NMI
TC1900008053.hg.1	13,23	13,86	-1,55	0,0163	0,7253	SAMD4B
TC1600009943.hg.1	8,73	9,36	-1,55	0,0195	0,7283	SEZ6L2
TC0700012242.hg.1	4,76	5,4	-1,55	0,0225	0,7417	C7orf66
TC1900008848.hg.1	5,98	6,62	-1,55	0,0237	0,7517	LILRA5
TSUnmapped000003	5,88	6,52	-1,55	0,0241	0,7517	NDUFA6
TC0700010198.hg.1	9,51	10,15	-1,55	0,0276	0,7679	FAM220A
TC0400009644.hg.1	6,87	7,5	-1,55	0,0288	0,7733	DUX4L7
TC1500010745.hg.1	5,3	5,93	-1,55	0,0306	0,7826	POLR2M
TC1900009186.hg.1	5,89	6,53	-1,55	0,0314	0,7831	ATP8B3
TC1300007221.hg.1	6,13	6,76	-1,55	0,0336	0,7831	SERPINE3
TC1300010007.hg.1	6,86	7,49	-1,55	0,0345	0,7863	TMEM255B
TC0600014360.hg.1	6,45	7,08	-1,55	0,0374	0,7908	IPCEF1
TC1100009412.hg.1	8,84	9,46	-1,54	0,0037	0,6181	PKNOX2
TC0900009746.hg.1	3,67	4,3	-1,54	0,0076	0,7253	IZUMO3
TC1400006723.hg.1	8,86	9,49	-1,54	0,0163	0,7253	REC8
TC1900008059.hg.1	12,29	12,91	-1,54	0,0175	0,7253	SUPT5H
TC0X00009727.hg.1	5,63	6,26	-1,54	0,0222	0,7371	SPANXN5
TC1500008727.hg.1	3,1	3,73	-1,54	0,0228	0,744	NDN
TC0100015287.hg.1	5,79	6,41	-1,54	0,0284	0,7719	FAM212B
TC1800007010.hg.1	5,77	6,39	-1,54	0,032	0,7831	DSG1
TC1500009120.hg.1	11,14	11,76	-1,54	0,0316	0,7831	RMDN3

TC1000008048.hg.1	10,09	10,71	-1,54	0,0363	0,7901	ZSWIM8
TC0700013330.hg.1	6,26	6,88	-1,54	0,0387	0,7977	AMZ1
TC1200011246.hg.1	4,44	5,06	-1,54	0,0394	0,8035	PTPRR
TC1000007461.hg.1	7,64	8,27	-1,54	0,0462	0,8056	RASSF4
TC0600007684.hg.1	12,36	12,99	-1,54	0,0473	0,8079	SLC39A7
TC0200012656.hg.1	5,58	6,2	-1,54	0,0491	0,8086	EFEMP1
TC0600012539.hg.1	6,8	7,41	-1,53	0,0097	0,7253	GABRR1
TC1000009420.hg.1	5,63	6,24	-1,53	0,0114	0,7253	INPP5A
TC0600008509.hg.1	8,96	9,57	-1,53	0,0092	0,7253	KCNQ5
TC0200008248.hg.1	8,76	9,38	-1,53	0,0186	0,7275	ELMOD3
TC0600006659.hg.1	9,32	9,94	-1,53	0,0219	0,7365	BPHL
TC1900011777.hg.1	11,99	12,6	-1,53	0,0206	0,7365	CYTH2
TC0900010800.hg.1	7,9	8,52	-1,53	0,0219	0,7365	ECM2
TC1400008382.hg.1	7,96	8,57	-1,53	0,0263	0,7579	TNFAIP2
TC0100008594.hg.1	12,55	13,17	-1,53	0,0283	0,7719	ROR1
TC2000009217.hg.1	6,64	7,25	-1,53	0,0318	0,7831	MATN4
TC0300008097.hg.1	4,4	5,02	-1,53	0,0338	0,7831	OR5H1
TC0300007074.hg.1	4,62	5,24	-1,53	0,0315	0,7831	SLC22A14
TC0200009060.hg.1	8,57	9,18	-1,53	0,0319	0,7831	STEAP3
TC1800009278.hg.1	6,38	6,99	-1,53	0,0335	0,7831	TCEB3CL
TC2200007576.hg.1	4,47	5,08	-1,53	0,0354	0,788	PARVG
TC0600012556.hg.1	5,45	6,06	-1,53	0,0362	0,7901	BACH2
TC0700009519.hg.1	5,01	5,62	-1,53	0,037	0,7901	OR2A25
TC0100015115.hg.1	6,05	6,67	-1,53	0,0376	0,7908	AMY1B; AMY1A
TC1000009851.hg.1	13,48	14,09	-1,53	0,0395	0,8036	FRMD4A
TC1300008963.hg.1	5,88	6,49	-1,53	0,0462	0,8056	DLEU7
TC1100012318.hg.1	3,59	4,21	-1,53	0,0452	0,8056	IL18
TC1100013066.hg.1	4,86	5,47	-1,53	0,043	0,8056	KRTAP5-10
TC2200008507.hg.1	4,91	5,52	-1,53	0,0422	0,8056	SLC5A4
TC1200008931.hg.1	6,72	7,33	-1,53	0,0495	0,8086	RITA1
TC1600010168.hg.1	10,02	10,62	-1,52	0,0036	0,6181	SHCBP1
TC1900011909.hg.1	10,54	11,15	-1,52	0,0079	0,7253	COPE
TC0500012947.hg.1	6,51	7,11	-1,52	0,0088	0,7253	GPRIN1
TC1000010744.hg.1	3,71	4,31	-1,52	0,0076	0,7253	TMEM26
TC0200006569.hg.1	5,88	6,49	-1,52	0,0187	0,7275	SOX11

TC1600006678.hg.1	5,35	5,96	-1,52	0,0208	0,7365	OR1F1
TC2200009345.hg.1	10,95	11,55	-1,52	0,0244	0,7517	C1QTNF6
TC0100009899.hg.1	9	9,6	-1,52	0,027	0,7652	C1orf54
TC1200012813.hg.1	8,48	9,08	-1,52	0,0278	0,768	B4GALNT1
TC1700006578.hg.1	4,14	4,75	-1,52	0,0281	0,7712	OR1A1
TC1600009105.hg.1	8,16	8,76	-1,52	0,0309	0,7831	ABCA3
TC0100015890.hg.1	6,22	6,83	-1,52	0,0326	0,7831	DENND4B
TC0X00006877.hg.1	4,22	4,83	-1,52	0,0357	0,788	MAGEB3
TC0100010009.hg.1	6,06	6,67	-1,52	0,039	0,8	LCE1D
TC1900009297.hg.1	6,05	6,65	-1,52	0,0397	0,8036	TBXA2R
TC0300012315.hg.1	9,88	10,48	-1,52	0,0409	0,8036	TPRA1
TC2200008055.hg.1	11,08	11,69	-1,52	0,0421	0,8056	PI4KA
TC0200011382.hg.1	7,48	8,08	-1,52	0,0486	0,8079	SNED1
TC1800007052.hg.1	4,48	5,08	-1,51	0,0081	0,7253	ASXL3
TC0900009897.hg.1	7,13	7,72	-1,51	0,0084	0,7253	FAM219A
TC0900006442.hg.1	8,4	9	-1,51	0,0157	0,7253	KANK1
TC0200008452.hg.1	5,64	6,23	-1,51	0,0083	0,7253	MAL
TC1000006907.hg.1	8,9	9,5	-1,51	0,0153	0,7253	STAM
TC0100015714.hg.1	10,59	11,18	-1,51	0,0107	0,7253	SV2A
TC1700012195.hg.1	4,98	5,57	-1,51	0,0107	0,7253	TBC1D26
TC0500010427.hg.1	10,95	11,54	-1,51	0,0112	0,7253	ZFR
TC1200010182.hg.1	8,94	9,54	-1,51	0,021	0,7365	BHLHE41
TC1100011157.hg.1	11,44	12,03	-1,51	0,0203	0,7365	PPP1R14B
TC1700007603.hg.1	7,75	8,34	-1,51	0,0242	0,7517	RASL10B
TC0700009243.hg.1	8,05	8,64	-1,51	0,0288	0,7733	TMEM140
TC0100011901.hg.1	8,92	9,52	-1,51	0,0314	0,7831	RAB4A; SPHAR
TC0600014259.hg.1	11,46	12,06	-1,51	0,0383	0,7949	ATP6V1G2-DDX39B
TC0300011997.hg.1	4,99	5,59	-1,51	0,0404	0,8036	BTLA
TC0100013038.hg.1	8,2	8,79	-1,51	0,0403	0,8036	FBXO42
TC0X00007999.hg.1	5,12	5,72	-1,51	0,0434	0,8056	BEX4
TC0500010553.hg.1	7,18	7,77	-1,51	0,0464	0,8056	FYB
TC0200008039.hg.1	5,3	5,89	-1,51	0,0483	0,8079	NOTO
TC0200008627.hg.1	13,71	14,31	-1,51	0,0489	0,8086	NPAS2
TC1600007986.hg.1	5,2	5,79	-1,5	0,0293	0,7785	NLRC5
TC0600014093.hg.1	8,46	9,05	-1,5	0,0367	0,7901	ZNRD1

TC1900008564.hg.1	6,4	6,99	-1,5	0,0396	0,8036	ATF5; MIR4751
TC1900011334.hg.1	4,22	4,81	-1,5	0,0459	0,8056	ZNF677
TC1600008137.hg.1	8,22	8,81	-1,5	0,0476	0,8079	CES3; CES2
TC0300008847.hg.1	8,1	7,51	1,5	0,0174	0,7253	ACKR4
TC0200011923.hg.1	4,78	4,19	1,5	0,0186	0,7275	APOB
TC0800012323.hg.1	7,07	6,49	1,5	0,0243	0,7517	CA13
TC0500011758.hg.1	5,29	4,7	1,5	0,0323	0,7831	CDO1
TC1700007108.hg.1	6,73	6,14	1,5	0,0333	0,7831	MIEF2
TC0X00007119.hg.1	4,03	3,45	1,5	0,0409	0,8036	JADE3
TC1100011196.hg.1	6,61	6,02	1,5	0,0415	0,8051	C11orf85
TC1800007859.hg.1	5,96	5,37	1,51	0,0081	0,7253	TYMSOS
TC0300012566.hg.1	4,87	4,27	1,51	0,0193	0,7283	A4GNT
TC1700008677.hg.1	5,18	4,58	1,51	0,0289	0,7736	CACNG4
TC1000012270.hg.1	8,47	7,88	1,51	0,0328	0,7831	EBF3
TC0200013669.hg.1	5,34	4,74	1,51	0,0465	0,8056	RFX8
TC0900007827.hg.1	5,09	4,49	1,51	0,043	0,8056	SPATA31E1
TC0800011249.hg.1	9,99	9,38	1,52	0,0071	0,7196	RNF19A
TC1000012511.hg.1	8,53	7,92	1,52	0,0169	0,7253	PRAP1
TC1500010909.hg.1	8,03	7,42	1,52	0,015	0,7253	STARD5
TC1100007630.hg.1	5,56	4,95	1,52	0,0194	0,7283	OR5T3
TC1200008275.hg.1	10,3	9,7	1,52	0,0208	0,7365	SYT1
TC0900007497.hg.1	6,24	5,64	1,52	0,0307	0,7826	TJP2
TC0100018488.hg.1	5,75	5,15	1,52	0,0313	0,7831	TMOD4
TC0700013342.hg.1	6,66	6,05	1,52	0,0401	0,8036	CHN2
TC1500008511.hg.1	12	11,39	1,52	0,0459	0,8056	LRRC28
TC1200012855.hg.1	6,47	5,86	1,53	0,0182	0,7275	VSIG10
TC1500007379.hg.1	9,13	8,52	1,53	0,0317	0,7831	FAM63B
TC1600010711.hg.1	12,39	11,77	1,53	0,0457	0,8056	CHTF8
TC0X00007015.hg.1	5	4,38	1,54	0,0085	0,7253	MED14OS
TC1100006925.hg.1	6,23	5,6	1,54	0,0121	0,7253	PDE3B
TC0700008657.hg.1	11,55	10,93	1,54	0,0186	0,7275	LRRC17
TC0900011955.hg.1	7,51	6,89	1,54	0,0211	0,7365	CAMSAP1
TC0600008670.hg.1	6,76	6,14	1,54	0,0212	0,7365	PRSS35
TC0500007154.hg.1	8,74	8,11	1,54	0,0218	0,7365	SLC1A3
TC1300008691.hg.1	8,56	7,94	1,54	0,0284	0,7719	FOXO1

TC0900009333.hg.1	11,87	11,24	1,54	0,0313	0,7831	FAM157B
TSUnmapped000005	5,1	4,47	1,54	0,0412	0,8036	SAG
TC0X00007370.hg.1	5,4	4,77	1,55	0,024	0,7517	APEX2
TC0900010069.hg.1	8,47	7,84	1,55	0,0256	0,7579	ZNF658B
TC1200008195.hg.1	6,58	5,93	1,56	0,0124	0,7253	LOC101928137; RP11-314D7.2
TC0400012458.hg.1	7,45	6,81	1,56	0,0184	0,7275	HPGD
TC0200009616.hg.1	9,18	8,53	1,56	0,0176	0,7275	KIF5C
TC1100012882.hg.1	6,85	6,21	1,56	0,0195	0,7283	OPCML
TC1700009398.hg.1	7,34	6,7	1,56	0,0244	0,7517	MIR22HG
TC2000008845.hg.1	10,46	9,81	1,56	0,027	0,7652	PLAGL2
TC0200008464.hg.1	10,4	9,76	1,56	0,0293	0,7785	KCNIP3
TC0600008571.hg.1	8,07	7,42	1,56	0,0319	0,7831	MYO6
TC1900007447.hg.1	8,85	8,2	1,56	0,0335	0,7831	ZNF101
TC0X00011319.hg.1	7,42	6,78	1,56	0,0377	0,7908	TMSB15B
TC0400009588.hg.1	4,6	3,94	1,57	0,0264	0,7593	ZFP42
TC0X00011353.hg.1	11,32	10,67	1,57	0,0343	0,7856	ASB9
TC2000008951.hg.1	10,34	9,69	1,57	0,0444	0,8056	GSS
TC1700006763.hg.1	5,96	5,3	1,58	0,0053	0,6757	ATP1B2
TC1800008418.hg.1	11,52	10,86	1,58	0,0131	0,7253	GAREM1
TC1500010768.hg.1	6,19	5,53	1,58	0,0241	0,7517	SCAMP5
TC0500011263.hg.1	7,82	7,15	1,58	0,0291	0,7757	SERINC5
TC1100006748.hg.1	6,19	5,53	1,58	0,0297	0,7826	OR2D3
TC1500009639.hg.1	11,04	10,38	1,58	0,0349	0,7875	ICE2
TC0700012896.hg.1	6,54	5,88	1,58	0,0401	0,8036	CTAGE6
TC0700008745.hg.1	9,34	8,68	1,59	0,0038	0,6193	PRKAR2B
TC0900007518.hg.1	5,88	5,21	1,59	0,011	0,7253	MAMDC2
TC1100006923.hg.1	9,2	8,52	1,59	0,014	0,7253	PDE3B
TC0X00009581.hg.1	10,26	9,59	1,59	0,0202	0,7365	ELK1
TC1100007470.hg.1	9,02	8,36	1,59	0,0339	0,7831	DDB2
TC0100015796.hg.1	10,32	9,63	1,6	0,0033	0,5994	POGZ
TC1200008703.hg.1	8,4	7,72	1,6	0,0173	0,7253	C12orf45
TC1600007023.hg.1	6,97	6,29	1,6	0,019	0,7283	ABCC1
TC0300006856.hg.1	10,01	9,33	1,6	0,026	0,7579	RARB
TC1400007244.hg.1	9,33	8,65	1,6	0,0335	0,7831	KTN1
TC0900011302.hg.1	5,76	5,08	1,6	0,0409	0,8036	TNFSF8

TC0900011394.hg.1	8,1	7,42	1,6	0,0457	0,8056	C5
TC1700010221.hg.1	7,21	6,53	1,6	0,045	0,8056	DHRS13
TC1200012161.hg.1	7,5	6,82	1,6	0,0428	0,8056	TRIAP1
TC0X00011014.hg.1	4,87	4,19	1,61	0,0366	0,7901	SPANXN2
TC1900011658.hg.1	8,06	7,38	1,61	0,0411	0,8036	TRAPPC5
TC2100008374.hg.1	5,29	4,59	1,62	0,003	0,5958	TSPEAR
TC1000012566.hg.1	11,67	10,98	1,62	0,0218	0,7365	MRPS16
TC0500009109.hg.1	5,06	4,36	1,62	0,0427	0,8056	GPX3
TC1700010639.hg.1	7,05	6,35	1,62	0,0418	0,8056	KRT10
TC0X00008264.hg.1	5,15	4,46	1,62	0,048	0,8079	AKAP14
TC0500008121.hg.1	10,73	10,03	1,62	0,0476	0,8079	ARSK
TC0400007713.hg.1	5,08	4,37	1,63	0,0111	0,7253	STAP1
TC1400007431.hg.1	6,8	6,09	1,63	0,0089	0,7253	SYNE2
TC1400008767.hg.1	10,71	10	1,64	0,0022	0,5304	STXBP6
TC0600011499.hg.1	7,15	6,43	1,64	0,0085	0,7253	HLA-DQB1
TC0X00011291.hg.1	7,14	6,42	1,64	0,0134	0,7253	PAGE2
TC0X00008120.hg.1	6,17	5,45	1,64	0,0084	0,7253	RGAG1
TC0600014361.hg.1	9,56	8,85	1,64	0,0186	0,7275	CNKSR3
TC0600007847.hg.1	8,06	7,34	1,64	0,0216	0,7365	CDKN1A
TC0200006725.hg.1	5,99	5,27	1,64	0,0206	0,7365	GREB1
TC0X00010559.hg.1	6,3	5,59	1,64	0,0223	0,7371	LRCH2
TC1100009398.hg.1	7,23	6,52	1,64	0,0297	0,7826	NRGN
TC1500007601.hg.1	12,35	11,64	1,64	0,0335	0,7831	HACD3
TC0600007820.hg.1	4,45	3,73	1,64	0,0387	0,7977	CLPSL1
TC0100013712.hg.1	4,89	4,19	1,64	0,0499	0,8086	EVA1B
TC0400007894.hg.1	4,69	3,97	1,65	0,003	0,5958	FAM47E; FAM47E-STBD1; STBD1
TC1200010292.hg.1	7,43	6,71	1,65	0,0096	0,7253	AMN1
TC0100007434.hg.1	10,12	9,39	1,65	0,0077	0,7253	EXTL1
TC0600009498.hg.1	11,83	11,11	1,65	0,0102	0,7253	EYA4
TC1100012126.hg.1	4,98	4,26	1,65	0,0116	0,7253	MMP8
TC1600011449.hg.1	8,09	7,36	1,65	0,0215	0,7365	SPIRE2
TC0300012339.hg.1	7,15	6,42	1,65	0,0242	0,7517	GATA2
TC1200010011.hg.1	8,98	8,26	1,65	0,0243	0,7517	HIST4H4
TC0600009336.hg.1	4,31	3,58	1,66	0,0054	0,6757	CLVS2
TC1300008626.hg.1	8,46	7,73	1,66	0,0064	0,6972	SMAD9

TC0100009273.hg.1	6,3	5,57	1,66	0,0136	0,7253	AMY2A
TC0300009661.hg.1	14,12	13,39	1,66	0,0118	0,7253	B3GNT5
TC1000011719.hg.1	5,54	4,81	1,66	0,0163	0,7253	CALHM1
TC1000008423.hg.1	9,86	9,13	1,66	0,013	0,7253	RPP30
TC0100011566.hg.1	5,61	4,88	1,66	0,0194	0,7283	PROX1
TC0X00010687.hg.1	8,14	7,41	1,66	0,0206	0,7365	CT47A7; CT47A5; CT47A6
TC1700009613.hg.1	6,43	5,7	1,66	0,0361	0,7901	ASGR2
TC0X00007937.hg.1	7,31	6,57	1,66	0,0453	0,8056	DRP2
TC1300009505.hg.1	11,61	10,87	1,67	0,0031	0,5994	ABCC4
TC0900008487.hg.1	6,3	5,56	1,67	0,006	0,6896	BSPRY
TC1300007727.hg.1	7,92	7,18	1,67	0,0127	0,7253	GPC6
TC1100007262.hg.1	5,48	4,74	1,67	0,0258	0,7579	EHF
TC0600011228.hg.1	9,61	8,88	1,67	0,033	0,7831	HIST1H2AK
TC1500009438.hg.1	6,55	5,81	1,67	0,0364	0,7901	LYSMD2
TC0900011611.hg.1	5,85	5,11	1,67	0,0445	0,8056	PIP5KL1
TC2100007140.hg.1	7,23	6,49	1,67	0,0471	0,8076	ETS2
TC0600008447.hg.1	6,56	5,81	1,68	0,0018	0,5304	ADGRB3
TC1000011056.hg.1	5,41	4,67	1,68	0,0054	0,6757	ADK
TC1000006707.hg.1	7,58	6,83	1,68	0,0071	0,7185	GATA3
TC0800011595.hg.1	11,32	10,58	1,68	0,0154	0,7253	EXT1; hunera
TC1200010006.hg.1	14,02	13,27	1,68	0,0091	0,7253	PLBD1
TC0300013949.hg.1	8,69	7,94	1,68	0,0118	0,7253	SATB1
TC1400010100.hg.1	10,35	9,61	1,68	0,0185	0,7275	ATG2B
TC1900009439.hg.1	6,4	5,66	1,68	0,0254	0,7579	CD70
TC0100007449.hg.1	12,49	11,75	1,68	0,0367	0,7901	SH3BGRL3
TC1400006933.hg.1	8,01	7,25	1,69	0,0072	0,7211	PAX9
TC1100012961.hg.1	6,64	5,88	1,69	0,015	0,7253	AMPD3
TC0500011260.hg.1	13,73	12,97	1,69	0,0191	0,7283	SERINC5
TC2000007083.hg.1	9,44	8,68	1,69	0,0219	0,7365	ID1
TC0500010779.hg.1	8,6	7,85	1,69	0,0208	0,7365	SLC38A9
TC0900009581.hg.1	8,61	7,86	1,69	0,0235	0,7517	FREM1
TC0800012377.hg.1	6,36	5,61	1,69	0,0242	0,7517	PPP1R16A
TC0100015795.hg.1	9,42	8,66	1,69	0,0325	0,7831	POGZ
TC1700008932.hg.1	5,69	4,92	1,7	0,0032	0,5994	ZACN
TC1900012056.hg.1	8,36	7,59	1,7	0,018	0,7275	ZNF772

TC0500013393.hg.1	5,07	4,3	1,7	0,0262	0,7579	CCNJL
TC0100013408.hg.1	9,55	8,79	1,7	0,0427	0,8056	GPN2
TC0400007868.hg.1	4,81	4,04	1,7	0,0481	0,8079	PARM1
TC0700012605.hg.1	10,74	9,97	1,71	0,005	0,6757	COPG2; TSGA13
TC0800012087.hg.1	10,77	10	1,71	0,0056	0,6757	LYNX1
TC1100009238.hg.1	7,01	6,24	1,71	0,0078	0,7253	HMBS
TC1200011675.hg.1	6,1	5,32	1,71	0,037	0,7901	GOLGA2P5
TC0800008146.hg.1	7,86	7,09	1,71	0,0477	0,8079	WWP1
TC0400007891.hg.1	7,4	6,62	1,72	0,0055	0,6757	ART3
TC0900012151.hg.1	6,93	6,15	1,72	0,0133	0,7253	ANKRD19P
TC0400009182.hg.1	5,33	4,55	1,72	0,0086	0,7253	01-mar
TC0200015529.hg.1	6,39	5,61	1,72	0,0164	0,7253	MDH1B
TC2000009458.hg.1	14,97	14,19	1,72	0,0131	0,7253	NFATC2
TC0800009865.hg.1	7,72	6,93	1,72	0,0121	0,7253	RP11-875O11.1; RHOBTB2
TC1500006902.hg.1	11,53	10,75	1,72	0,0181	0,7275	FAM98B
TC0X00010682.hg.1	8	7,22	1,72	0,0356	0,788	CT47B1
TC1200009847.hg.1	13,87	13,08	1,73	0,0074	0,7211	A2M
TC0100006761.hg.1	10,8	10,01	1,73	0,0088	0,7253	CA6
TC1300009488.hg.1	6,31	5,51	1,73	0,0156	0,7253	DCT
TC1400010592.hg.1	10,62	9,82	1,73	0,0143	0,7253	NUBPL
TC0500007376.hg.1	9,04	8,23	1,74	0,0021	0,5304	FST
TC1900011721.hg.1	10,25	9,45	1,74	0,0168	0,7253	HKR1
TC2100006980.hg.1	7,16	6,36	1,74	0,0157	0,7253	ITSN1
TC0400007285.hg.1	7,24	6,44	1,74	0,0115	0,7253	LIAS
TC1100013108.hg.1	5,28	4,48	1,74	0,0351	0,7875	GRAMD1B
TC0200015128.hg.1	8,33	7,53	1,74	0,0382	0,7949	PDE1A
TC1200007804.hg.1	7,96	7,16	1,74	0,0423	0,8056	METTL7B
TC1100010440.hg.1	9,98	9,17	1,75	0,0012	0,4402	MPPED2
TC0100007638.hg.1	11,98	11,17	1,75	0,0023	0,5304	SERINC2
TC1500009702.hg.1	10,96	10,15	1,75	0,0091	0,7253	RPS27L
TC0300009247.hg.1	9,38	8,58	1,75	0,0217	0,7365	ARHGEF26
TC0600009243.hg.1	4,97	4,16	1,75	0,0448	0,8056	RFX6
TC0300011849.hg.1	14,03	13,22	1,76	0,0038	0,6193	ABI3BP
TC0100015333.hg.1	5,95	5,13	1,76	0,0171	0,7253	PTPN22
TC0800006927.hg.1	9,43	8,61	1,77	0,0035	0,6181	LPL

TC0500007077.hg.1	5,69	4,86	1,77	0,0067	0,7094	NPR3
TC0600012863.hg.1	11,83	11	1,77	0,0247	0,7537	REV3L
TC0100017948.hg.1	12,46	11,62	1,78	0,0024	0,5572	CHML
TC1100009917.hg.1	7,09	6,26	1,78	0,0173	0,7253	HBB
TC1400006859.hg.1	10,8	9,97	1,78	0,0093	0,7253	NPAS3
TC0600011971.hg.1	7,55	6,7	1,79	0,0115	0,7253	PTCHD4
TC0700013368.hg.1	9,69	8,85	1,79	0,0119	0,7253	ZNF736
TC1900011741.hg.1	7,29	6,46	1,79	0,0261	0,7579	CEACAM5
TC1200010837.hg.1	4,21	3,37	1,79	0,0484	0,8079	GPR84
TC0500008342.hg.1	10,92	10,07	1,8	0,0336	0,7831	DCP2
TC0X00007713.hg.1	9,66	8,81	1,81	0,007	0,7181	LPAR4
TC0300008672.hg.1	4,02	3,16	1,81	0,0121	0,7253	CFAP100
TC1500010946.hg.1	7,06	6,21	1,81	0,0333	0,7831	TM2D3
TC0600012329.hg.1	6,29	5,42	1,82	0,0054	0,6757	IMPG1
TC1100012389.hg.1	12,81	11,95	1,82	0,0139	0,7253	CADM1
TC0200009537.hg.1	7,86	7	1,82	0,0151	0,7253	KYNU
TC1500008120.hg.1	4,9	4,04	1,82	0,0152	0,7253	SH3GL3
TC2000009945.hg.1	5,39	4,53	1,83	0,0101	0,7253	FAM209A
TC1500009355.hg.1	7,12	6,25	1,83	0,0085	0,7253	SHC4
TC1100008518.hg.1	10,56	9,68	1,83	0,0227	0,744	ACER3
TC0500013384.hg.1	11,16	10,28	1,84	0,0012	0,4402	PPP2R2B
TC1300007718.hg.1	15,26	14,38	1,84	0,0069	0,7181	GPC6
TC1400009345.hg.1	7,99	7,1	1,85	0,0026	0,5632	C14orf39
TC0100010543.hg.1	12,92	12,04	1,85	0,0032	0,5994	ATP1B1
TC0500012367.hg.1	6,08	5,2	1,85	0,0129	0,7253	GRXCR2
TC1200009852.hg.1	9,52	8,63	1,85	0,0316	0,7831	PZP
TC0100008618.hg.1	9,33	8,43	1,86	0,0054	0,6757	AK4
TC0100008183.hg.1	5,66	4,76	1,86	0,0204	0,7365	CYP4Z1
TC1100008034.hg.1	8,7	7,81	1,86	0,0442	0,8056	KAT5
TC1700011101.hg.1	6,55	5,64	1,87	0,0421	0,8056	CHAD
TC0200015645.hg.1	4,74	3,82	1,88	0,0144	0,7253	ABCA12
TC0X00011277.hg.1	13,32	12,41	1,88	0,017	0,7253	CA5B
TC0300013858.hg.1	9,7	8,78	1,89	0,0012	0,443	CD200; RP11-90K6.1
TC2000009918.hg.1	6,55	5,63	1,89	0,0101	0,7253	BPI
TC0X00010442.hg.1	7,52	6,6	1,89	0,0116	0,7253	TMSB15B; RP11-722G7.1

TC0500013227.hg.1	7,26	6,34	1,89	0,0301	0,7826	CDC42SE2
TC0600011503.hg.1	9,21	8,29	1,89	0,0393	0,8031	HLA-DQB2
TC0400011685.hg.1	8,6	7,67	1,91	0,0173	0,7253	PRSS12
TC1200007630.hg.1	10,61	9,67	1,92	0,0116	0,7253	LETMD1
TC1200010101.hg.1	7,89	6,94	1,93	0,006	0,6896	SLCO1A2
TC1100011247.hg.1	11,12	10,16	1,94	0,0054	0,6757	RNASEH2C
TC1100010505.hg.1	10,31	9,34	1,95	0,0214	0,7365	ABTB2
TC0700011584.hg.1	8,27	7,3	1,96	0,0006	0,3399	FGL2
TC1200008116.hg.1	13,95	12,98	1,96	0,0365	0,7901	MDM2
TC1700010604.hg.1	9,18	8,2	1,97	0,0046	0,6723	NR1D1
TC1500007691.hg.1	11,67	10,69	1,97	0,0088	0,7253	GLCE
TC1500010160.hg.1	8,01	7,02	1,98	0,0166	0,7253	CTSH
TC0200011890.hg.1	6,97	5,98	1,98	0,0216	0,7365	LAPTM4A
TC0700011675.hg.1	9,68	8,69	1,99	0,0062	0,6924	SEMA3D
TC0900012033.hg.1	11,14	10,14	1,99	0,0065	0,6972	DPP7
TC0700008584.hg.1	8,52	7,53	1,99	0,0149	0,7253	AP1S1
TC0X00008054.hg.1	8,63	7,64	1,99	0,011	0,7253	RNF128
TC0X00008468.hg.1	6,1	5,11	1,99	0,0406	0,8036	CCDC160
TC0800012312.hg.1	10,44	9,44	2	0,0005	0,321	SGK3
TC0X00011393.hg.1	8,14	7,14	2	0,0059	0,6896	CT47A5; CT47A2; CT47A1
TC0900008811.hg.1	13,69	12,69	2	0,0091	0,7253	STXBP1
TC1600007819.hg.1	7,66	6,66	2	0,0247	0,7537	NKD1
TC0300013513.hg.1	9,93	8,93	2	0,0456	0,8056	P3H2
TC0800011881.hg.1	10,26	9,25	2,01	0,0021	0,5304	NDRG1
TC0400010171.hg.1	7,2	6,19	2,01	0,0192	0,7283	LCORL
TC0200014775.hg.1	4,87	3,85	2,02	0,0073	0,7211	KCNH7
TC1400009973.hg.1	8,27	7,26	2,02	0,0191	0,7283	RPS6KA5
TC0700009222.hg.1	8,65	7,63	2,03	0,0053	0,6757	LRGUK
TC0600011133.hg.1	4,18	3,15	2,04	0,0045	0,6694	HIST1H2BE
TC0500009046.hg.1	5,49	4,45	2,05	0,0396	0,8036	ADRB2
TC1400010733.hg.1	7,85	6,8	2,07	0,026	0,7579	SFTA3
TC0X00006938.hg.1	8,94	7,88	2,08	0,0015	0,4877	CFAP47
TC1300006919.hg.1	10,37	9,31	2,08	0,0056	0,6757	FREM2
TC0500011520.hg.1	6,02	4,96	2,08	0,0133	0,7253	ERAP1
TC0700007480.hg.1	6,92	5,86	2,08	0,0312	0,7831	ABCA13

TC0400010596.hg.1	5,19	4,13	2,09	0,0019	0,5304	GABRG1
TC0700008927.hg.1	6,93	5,86	2,09	0,0117	0,7253	ING3
TC1200008133.hg.1	9,16	8,1	2,09	0,011	0,7253	LYZ
TC1700007014.hg.1	9,32	8,25	2,1	0,0458	0,8056	UBB
TC1300008091.hg.1	7,07	5,99	2,11	0,0022	0,5304	ATP11A
TC1400007769.hg.1	7,91	6,81	2,13	0,0074	0,7211	ADCK1
TC1100007995.hg.1	7,05	5,95	2,13	0,0196	0,7295	CDCA5
TC0600011124.hg.1	12,79	11,7	2,13	0,0222	0,7371	HIST1H3B
TC0800010825.hg.1	6,53	5,42	2,15	0,0181	0,7275	JPH1
TC1600007368.hg.1	8,52	7,4	2,16	0,0442	0,8056	ATP2A1
TC0900007618.hg.1	7,96	6,85	2,17	0,0015	0,4877	PCSK5
TC0100009395.hg.1	4,85	3,73	2,18	0,001	0,4082	CD53
TC0300008050.hg.1	5,93	4,8	2,18	0,0059	0,6896	NSUN3
TC0200010502.hg.1	12,62	11,49	2,19	0,0046	0,6723	FAM117B
TC1000008388.hg.1	8,54	7,41	2,2	0,0002	0,2037	FAS
TC1200009926.hg.1	8,2	7,06	2,21	0,014	0,7253	TAS2R50
TC0800007221.hg.1	7,77	6,62	2,22	0,0026	0,5632	NRG1
TC1300008708.hg.1	8,48	7,33	2,23	0,0181	0,7275	ELF1
TSUnmapped000002	5,49	4,33	2,24	0,0002	0,2037	MLXIP
TC1100012269.hg.1	10,44	9,25	2,27	0,0002	0,1896	ARHGAP20
TC1000011774.hg.1	9,14	7,95	2,28	0,0027	0,5699	SORCS1
TC0700013347.hg.1	13,33	12,13	2,29	0,0003	0,2037	AQP1
TC0500012149.hg.1	7,19	6	2,29	0,0051	0,6757	KLHL3
TC0500010924.hg.1	8,98	7,77	2,32	0,0006	0,3213	ADAMTS6
TC1300009858.hg.1	7,08	5,86	2,32	0,0016	0,491	TUBGCP3
TC0800008330.hg.1	8,57	7,36	2,32	0,0022	0,5304	POP1
TC0400012212.hg.1	5,4	4,18	2,33	0,042	0,8056	ASIC5
TC0X00007034.hg.1	8,55	7,33	2,33	0,0494	0,8086	NYX
TC0100011661.hg.1	6,51	5,28	2,34	0,0003	0,2037	MARK1
TC1000007776.hg.1	9,44	8,18	2,4	0,0057	0,6757	ZNF365
TC0500012666.hg.1	11,37	10,1	2,41	0,0002	0,1952	ATP10B
TC1500010796.hg.1	14,42	13,14	2,43	0,0021	0,5304	SYNM
TC1200011063.hg.1	8,53	7,24	2,44	0,0032	0,5994	AVPR1A
TC1800009212.hg.1	7,82	6,54	2,44	0,0065	0,6972	PARD6G
TC0200016073.hg.1	9,73	8,44	2,46	0,0003	0,2037	KCNJ13

TC0100007645.hg.1	8,33	7,01	2,5	0,0003	0,2037	TINAGL1
TC0200010640.hg.1	9,35	8,02	2,53	7,74E-05	0,1508	UNC80
TC0300007748.hg.1	10,47	9,1	2,59	0,0021	0,5304	MITF
TC1500009861.hg.1	7,46	6,08	2,6	0,0144	0,7253	ITGA11
TC0900011209.hg.1	6,2	4,82	2,6	0,022	0,7365	C9orf84
TC0900011192.hg.1	9,88	8,49	2,63	0,0009	0,4068	LPAR1
TC0600006873.hg.1	9,33	7,87	2,77	8,75E-05	0,1561	BMP6
TC1700010997.hg.1	8,41	6,94	2,78	5,52E-05	0,1432	HOXB9
TC1000011599.hg.1	11,86	10,35	2,84	3,09E-05	0,1105	CPN1
TC0700008873.hg.1	10,76	9,19	2,96	0,001	0,4137	CAV1
TC0700011658.hg.1	8,14	6,43	3,28	7,43E-05	0,1508	SEMA3E
TC1700009745.hg.1	8,2	6,47	3,31	0,0142	0,7253	MYH2
TC0X00009917.hg.1	10,12	8,37	3,37	0,0008	0,4006	EDA2R
TC0100016218.hg.1	11,07	9,27	3,47	0,0002	0,2037	RGS5
TC1800008828.hg.1	6,29	4,41	3,67	0,0043	0,6489	CCBE1
TC0200015771.hg.1	9,57	7,67	3,74	0,0406	0,8036	TUBA4A
TC0700007479.hg.1	10,71	8,64	4,22	3,61E-06	0,026	ABCA13
TC0100015636.hg.1	12	9,89	4,32	2,18E-06	0,026	GJA5
TC0400007782.hg.1	8,92	6,73	4,56	0,0001	0,1896	MUC7
